# Betalain biosynthesis in red pulp pitaya is regulated via HuMYB132: a R-R type MYB transcription factor

**DOI:** 10.1186/s12870-023-04049-6

**Published:** 2023-01-13

**Authors:** Fangfang Xie, Canbin Chen, Jiayi Chen, Jiaxuan Chen, Qingzhu Hua, Kamran Shah, Zhike Zhang, Jietang Zhao, Guibing Hu, Jianye Chen, Yonghua Qin

**Affiliations:** 1 Guangdong Provincial Key Laboratory of Postharvest Science of Fruits and Vegetables/Key Laboratory of Biology and Genetic Improvement of Horticultural Crops (South China), Ministry of Agriculture and Rural Affairs, College of Horticulture, South China Agricultural University, Guangzhou, 510642 China; 2 College of Agriculture, Guangxi University, Nanning, 530004 China

**Keywords:** Betalain biosynthesis, MYB transcription factor, Pitaya, Transcription activity

## Abstract

**Background:**

Multiple MYB transcription factors (TFs) are involved in the regulation of plant coloring. Betalain is a kind of natural plant pigment and its biosynthesis is regulated by a number of enzymes. Despite this, little is known about the molecular properties and roles of MYB TFs in pitaya betalain biosynthesis.

**Results:**

In the present study, we identified a 1R-MYB gene, *HuMYB132*, which is preferentially expressed in red-pulp pitaya at the mature stage. It was clustered with Arabidopsis R-R-type genes and had two DNA-binding domains and a histidine-rich region. The expression assays in *N*. *benthamiana* and yeast indicated that HuMYB132 is a nucleus-localized protein with transcriptional activation activity. Dual luciferase reporter assay and electrophoretic mobility shift assays (EMSA) demonstrated that *HuMYB132* could promote the transcriptional activities of *HuADH1*, *HuCYP76AD1–1*, and *HuDODA1* by binding to their promoters. Silencing *HuMYB132* reduced betalain accumulation and the expression levels of betalain biosynthetic genes in pitaya pulps.

**Conclusions:**

According to our findings, *HuMYB132*, a R-R type member of 1R-MYB TF subfamily, positively regulates pitaya betalain biosynthesis by regulating the expression of *HuADH1*, *HuCYP76AD1–1*, and *HuDODA1*. The present study provides a new theoretical reference for the management of pitaya betalain biosynthesis and also provides an essential basis for future regulation of betalain biosynthesis in *Hylocereus.*

**Supplementary Information:**

The online version contains supplementary material available at 10.1186/s12870-023-04049-6.

## Background

Transcription factors (TFs) play important roles in plant growth and development. Based on the pitaya genome and transcriptome data, a comprehensive regulatory network of betalain biosynthesis in pitaya fruit was constructed to provide multiple potential candidate TFs in the pitaya betalain biosynthesis [1]. MYB TFs are among the most abundant plant-specific TFs which are classified into four subfamilies according to the presence of one to four highly conserved MYB repeats: MYB-related (1R-MYB), R2R3-MYB (2R-MYB), R1R2R3-MYB (3R-MYB), and 4R-MYB [2]. Surprisingly, the pitaya genome contains 75 1R-MYB, 105 R2R3-MYB, four R1R2R3-MYB, and one 4R-MYB TFs. *HuMYB1* is an R2R3-MYB negative regulator of pitaya betalain biosynthesis that inhibits the transcription activities of *HuADH1*, *HuCYP76AD1–1*, and *HuDODA1* [3]. Furthermore, an inventory of the Arabidopsis genome reveals that it has 126 R2R3-MYB, five R1R2R3-MYB, 64 MYB-like, and three atypical MYB genes [4]. Many *3R-MYB* and *2R-MYB* genes have been extensively studied in *A. thaliana*, giving us a better understanding of MYB gene functions in other plant species [5]. For instance, a homologous R2R3-MYB TF of *AtPAP1/2* in beet, *BvMYB1*, enhanced betalain biosynthesis by activating the expression levels of *BvCYP76AD1* and *BvDODA1* [6]. However, the identification and role of other MYB TFs regulating betalain biosynthesis remains largely unknown.

Pitaya is a popular tropical fruit belonging to the *Hylocereus* and *Seleniereus* in the Cactaceae family of the Caryophyllales order. According to peel and pulp color, pitayas are mainly categorized into three species: *H. undatus* (red peel with white pulp), *H. monacanthus* or *H. polyrhizus* (red peel with red pulp), and *H. megalanthus* or *S. megalanthus* (yellow peel with white pulp) [7]. Pitaya is the only commercial cultivation of fruit containing abundant betalains for consumers. Betalain biosynthesis and accumulation are responsible for different peel and pulp colors in pitaya. Interestingly, betalains are restricted to the angiosperm order of core Caryophyllales, with the exception of Caryophyllaceae, Molluginaceae, Kewaceae, Limeaceae, Macarthuriaceae, and Simmondsiaceae families, which have been reported to produce anthocyanins [8]. Betalains and anthocyanins have never been found in the same plant species and share a mutual exclusion relationship [9]. Interestingly, the precursors of betalains and anthocyanins are Tyr and Phe, respectively, and both of them are synthesized from arogenate [10, 11]. Moreover, a recent study inferred that anthocyanins in most plants of Caryophyllales were replaced by betalains, probably due to betalain biosynthetic genes, i.e., *ADH*, *CYP76AD*, and *DODA* happening in several evolutionary events [12]. However, the mechanism of the mutual exclusion between betalains and anthocyanins still remains unknown.

Betalains are vacuole-localized, water-soluble, nitrogen-containing, and tyrosine-derived pigments which are divided into purple-red betacyanins and yellow betaxanthins [9]. Three key enzymatic reactions have been characterized, including the tyrosine biosynthesis by arogenate dehydrogenase (ADH) [10], the betalamic acid biosynthesis by 4,5-DOPA extradiol dioxygenase (DODA) [13] and the cyclo-DOPA synthesis by a cytochrome P450 enzyme (CYP76AD1) [14], which were critical for betalain biosynthesis. Betacyanins are synthesized via glucosyltransferase (GT) and acyltransferase (ATs), while betaxanthins are produced through spontaneous reactions [15–18]. So far, candidate genes, proteins and metabolites of betalain biosynthesis were isolated in green-, red-, and yellow-peeled pitayas based on microRNAs, transcriptomics, proteomics, and metabolomics analyses [19–23].

Our previous studies suggested that betalain-related structural genes, i.e. *HuADH1*, *HuCYP76AD1–1*, and *HuDODA1* were gathered with other Caryophyllales *ADHα*, *CYP76AD1α*, and *DODAα* genes, respectively, on the same chromosome in pitaya genome [3]. Moreover, a predicted R-R-type member of 1R-MYB TF, *HuMYB132* (*HU04G01397.1*), showed a co-expression pattern relationship with betalain biosynthesis genes by weighted correlation network analysis (WGCNA) [1, 3]. As a result, this study was designed for the first time to elucidate the molecular mechanism of *HuMYB132* TF which was further explored to be responsible for regulating the expression of betalain biosynthesis genes of *HuADH1*, *HuCYP76AD1–1*, and *HuDODA1*. Our findings could shed light on the regulatory mechanism of 1R-MYB in pitaya betalain biosynthesis.

## Results

### Identification of *HuADH1*, *HuCYP76AD1–1*, and *HuDODA1* involved in pitaya Betalain biosynthesis

**Fig. 1:**
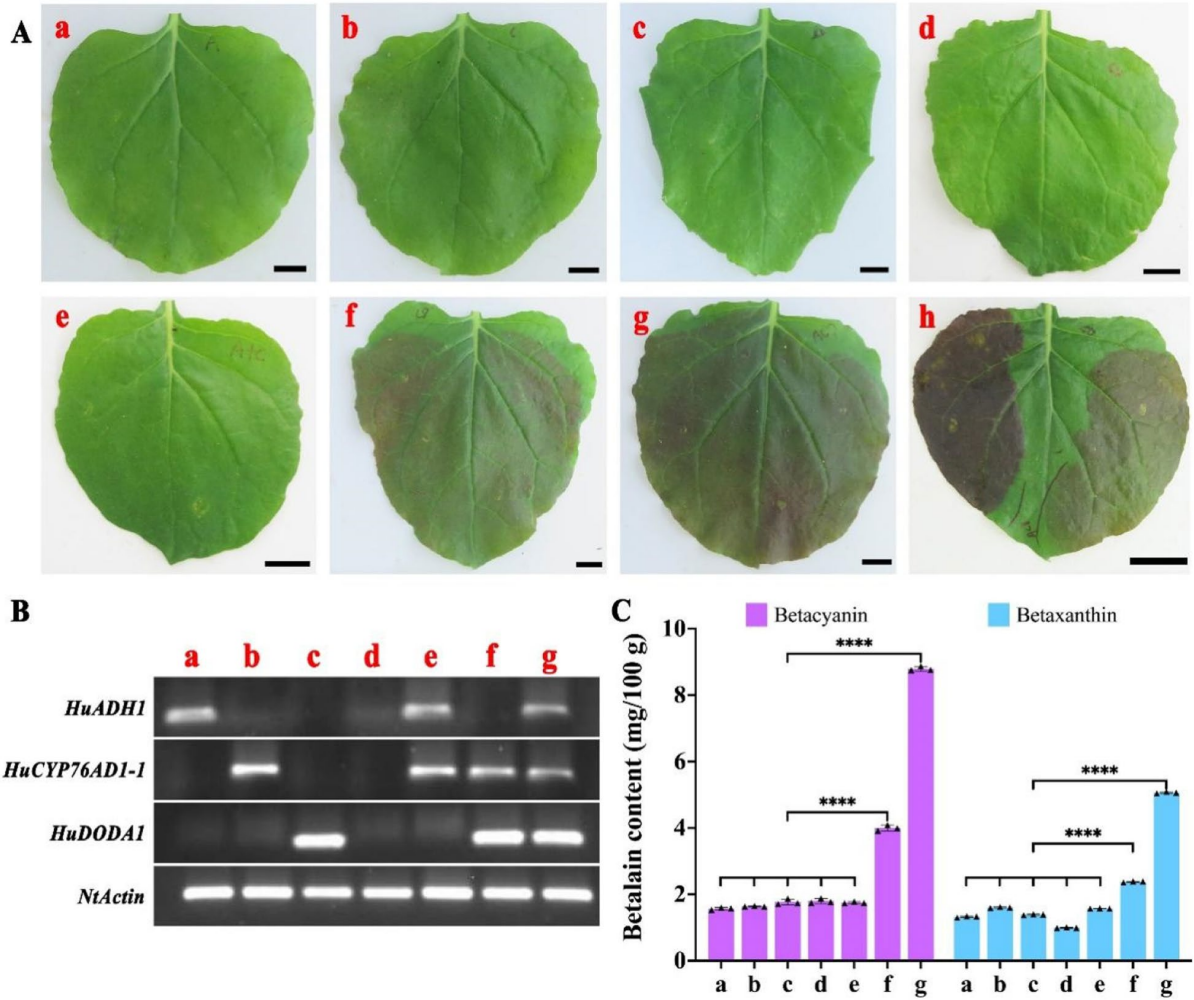
The transient expressions of *HuADH1*, *HuCYP76AD1–1*, and *HuDODA1* in *N. benthamiana*. **A**, The phenotypes of *N. benthamiana* leaves after infiltration. (a) *HuADH1*, (b) *HuCYP76AD1–1*, (c) *HuDODA1*, (d) Empty vector, (e) *HuADH1* + *HuCYP76AD1–1*, (f) *HuCYP76AD1–1* + *HuDODA1,* (g) *HuADH1* + *HuCYP76AD1–1* + *HuDODA1*, (h) Comparison of *HuADH1* + *HuCYP76AD1–1* (right) and *HuADH1* + *HuCYP76AD1–1* + *HuDODA1* (left) in the same *N. benthamiana* leaf. **B**, The RT-PCR analyses of infiltrated leaves. **C**, The betacyanin and betaxanthin content of infiltrated leaves. Small triangles represent the distribution of data for each biological replicate. Results were expressed as means ± SD (*n* = 3), with significance values presented as: * *p* < 0.05; ** *p* < 0.01; *** *p* < 0.001; **** *p* < 0.0001; while non-significant (ns) (*p* > 0.05)

Our previous studies showed that *HuADH1*, *HuCYP76AD1–1*, and *HuDODA1* were respectively gathered with *ADHα*, *CYP76AD1α,* and *DODAα* genes and their expression levels in the pulp of *H. monacanthus* (red peel with red pulp) were significantly higher than those of *H. undatus* (red peel with white pulp) [1]. *HuADH1*, *HuCYP76AD1–1*, and *HuDODA1* are co-localized on chromosome 3 according to the *H. undatus* genome data. A transient expression assay was performed in *N. benthamiana* leaves to elucidate the roles of *HuADH1*, *HuCYP76AD1–1*, and *HuDODA1* in betalain biosynthesis. As shown in Fig. 1 (Additional file 1: Fig. S1), no betalain accumulation was detected in *N. benthamiana* leaves when *HuADH1*, *HuCYP76AD1–1*, and *HuDODA1* were individually overexpressed, which is consistent with the phenomenon of the empty vector. Co-infiltration of *A. tumefaciens* containing *HuCYP76AD1–1* and *HuDODA1* resulted in the production of betalains compared to no visible pigmentation for co-expression of *HuADH1* and *HuCYP76AD1–1*. Betalain contents in *N. benthamiana* leaves of co-expression of *HuADH1*, *HuCYP76AD1–1*, and *HuDODA1* were two times higher than that of co-expression of *HuCYP76AD1–1* and *HuDODA1*. These results suggested that *HuCYP76AD1–1* and *HuDODA1* are key structural genes for betalain biosynthesis in pitaya, while *HuADH1* is a key structural gene for promoting betalain accumulation.

### *HuMYB132* exhibits a preferentially expression level in red-pulp pitaya at mature stage

**Fig. 2:**
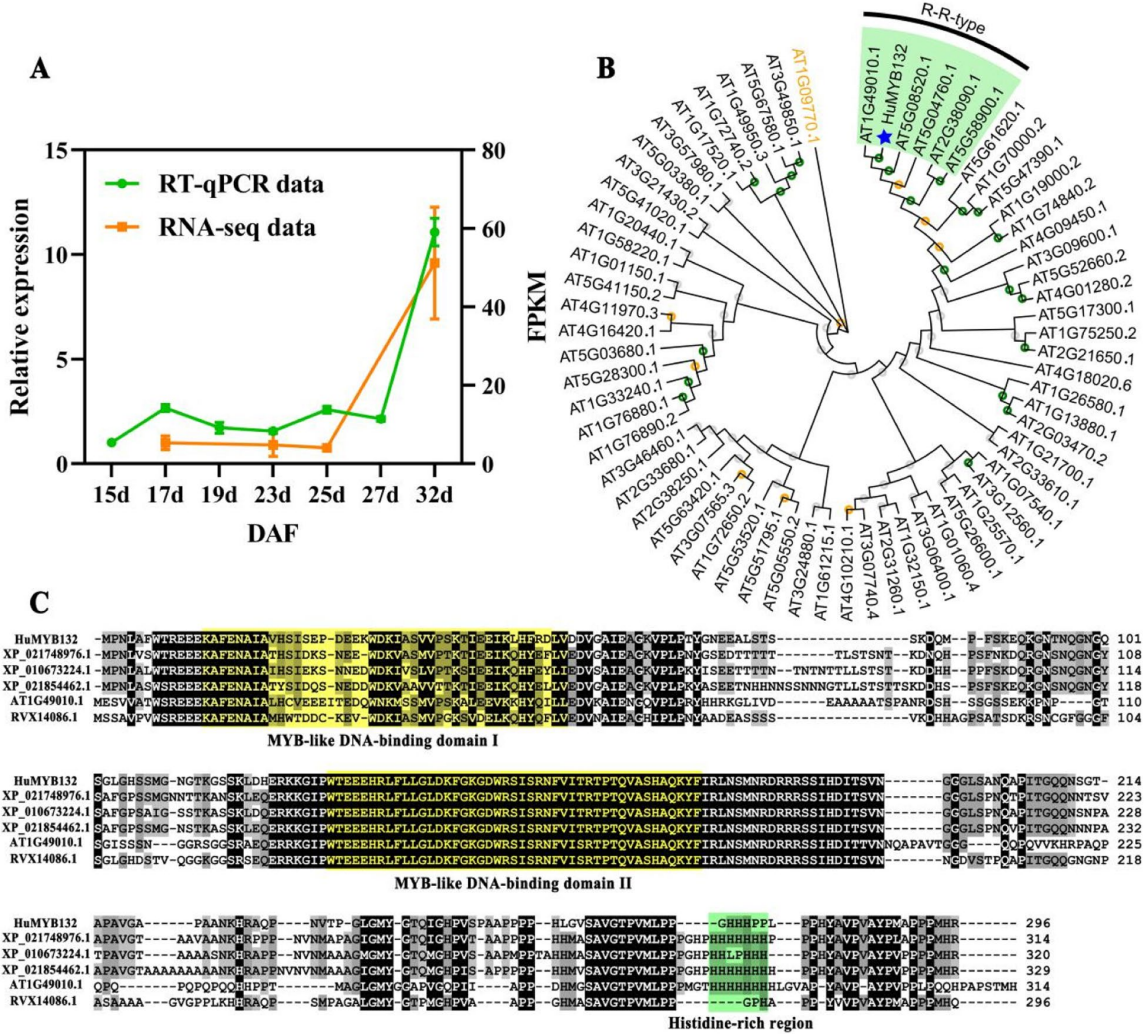
The expression and sequence analysis of *HuMYB132*. **A**, The expression patterns of *HuMYB132* during fruit developmental stages in ‘Guanhuahong’ pitaya. Data represents the mean values from three biological replicates (±S.D.). **B**, The phylogenetic analyses of *HuMYB132*, *AT1G09770.1* (an ‘unusual’ *MYB* gene) and 64 Arabidopsis *1R-MYB* genes were performed in MEGA7.0 software with 1000 replications of ML method. Green background indicates R-R-type genes. *HuMYB132* was labelled with a blue star. **C**, The sequence alignment analyses of *HuMYB132* and homologous genes from the other plants. *Chenopodium quinoa*, *XP_021748976.1*; *Beta vulgaris*, *XP_010673224.1*; *Spinacia oleracea*, *XP_021854462.1*; *Arabidopsis thaliana*, *AT1G49010.1*; *Vitis vinifera*, *RVX14086.1*. Yellow and green backgrounds indicate DBD domains and histidine-rich regions, respectively

Based on the WGCNA of pitaya transcriptome data, a 1R-MYB gene named *HuMYB132* was obtained, and it was predominantly expressed in the mature fruit of red-pulp pitaya [1, 3]. The highest expression level of *HuMYB132* was detected in mature fruit (32 d), which is consistent with RNA-Seq data during pitaya fruit development (Fig. 2A). *HuMYB132* belonged to the R-R-type MYB TFs and was phylogenetically related to *AT1G49010.1* (*AtMYBL*) involving in abiotic stress (Fig. 2B) [24]. It encoded 296 amino acid residues with a theoretical pI of 9 and a molecular weight of 32 kDa, as well as two DNA binding domains (DBD) (named DBD I and DBD II) at the N-terminal and a histidine-rich region at the C-terminal (Fig. 2C). Based on these results, it seems that *HuMYB132* is a candidate 1R-MYB TF responsible for betalain biosynthesis in pitaya.

### HuMYB132 is a nuclear protein with transcriptional activation activity

**Fig. 3:**
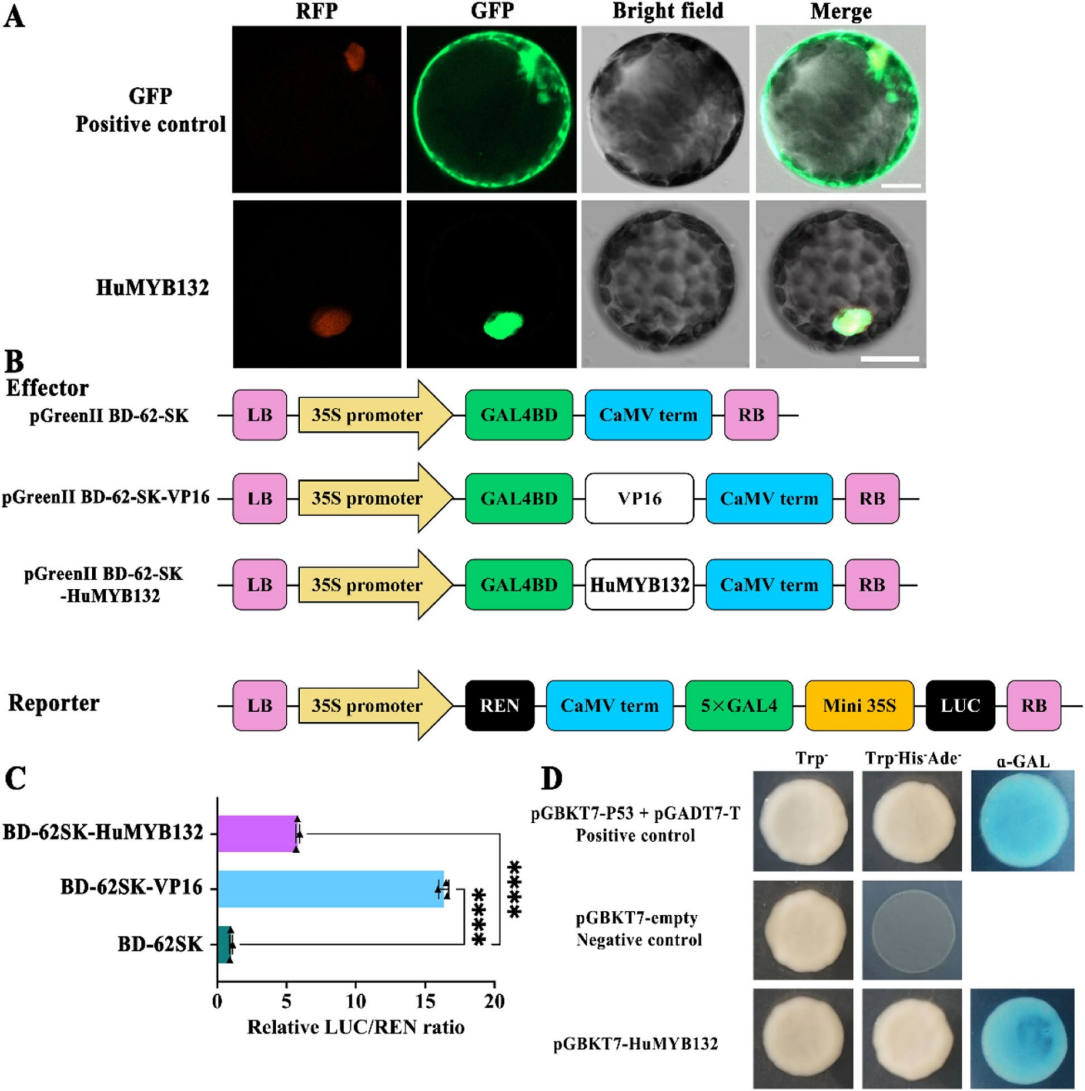
*HuMYB132* is a nucleus-localized transcription activator. **A**, Subcellular localization of *HuMYB132* in the protoplast of *N. benthamiana* leaves. Bars = 20 μm. **B**, Diagrams of the reporter and effector vectors. **C**, Transcriptional activation of *HuMYB132* in *N. benthamiana* leaves. The LUC/REN ratio of the empty BD-62SK vector was used as a calibrator (set as 1) while BD-62SK-VP16 was a positive control. Small triangles represent the distribution of data for each biological replicate. Results were expressed as means ± SD (n = 3), with significance values presented as: **** p < 0.0001. **D**, Transcriptional activation of *HuMYB132* in yeast cells. pGBKT7 and pGBKT7–53+ pGADT7-T were used as negative and positive controls, respectively

To investigate the subcellular localization of *HuMYB132*, its encoding region was fused with the *GFP* gene under the control of the CaMV promoter. The GFP-HuMYB132 fluorescence was exclusively observed in the nucleus of *N. benthamiana* protoplasts while the positive control fluorescence was dispersed throughout the cytosol and nucleus (Fig. 3A). These results suggested that HuMYB132 is a nuclear-localized protein.

To explore whether *HuMYB132* has transcriptional activation activity, the full-length cDNA was fused to the GAL4BD domain of the BD-62SK vector as an effector and transiently expressed in *N. benthamiana* leaves with reporter (Fig. 3B). The BD-62SK-HuMYB132 and positive control (BD-62SK-VP16) showed a significantly higher value of LUC/REN ratio than the negative control (BD-62SK) (Fig. 3C). Besides, full-length cDNA of *HuMYB132* was fused to the GAL4BD domain of the pGBKT7 vector and introduced into yeast cells. Compared with negative control (pGBKT7), the transformed yeast cells of pGBKT7-HuMYB132 and positive control (pGBKT7-P53 + pGADT7-T) grew well on SD/−Trp-His-Ade medium and showed α-Gal activity (Fig. 3D). These results suggested that HuMYB132 has transcriptional activation activity.

### HuMYB132 directly binds to the promoters of Betalain biosynthesis genes and activates their transcription

**Fig. 4:**
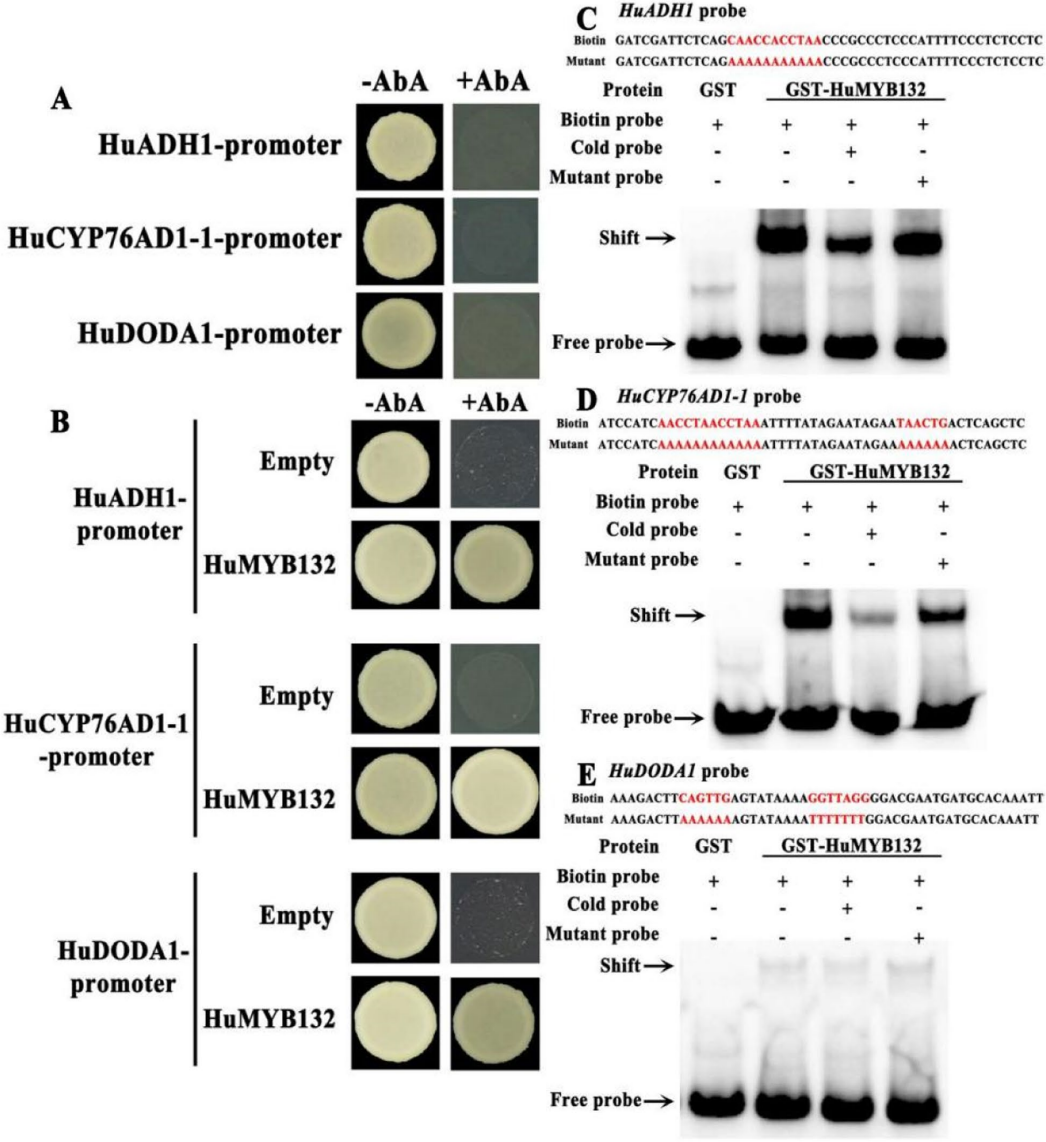
*HuMYB132* directly binds to the promoters of *HuADH1*, *HuCYP76AD1–1*, and *HuDODA1* according to Y1H and EMSA assays. **A**, Bait yeasts with the promoter sequences *HuADH1*, *HuCYP76AD1–1* and *HuDODA1* were cultured on synthetic dropout medium lacking Ura (SD/−Ura) supplemented with 200 ng/mL AbA concentration. **B**, The interaction of *HuMYB132* and bait yeasts was determined by yeast growth on SD/−Leu medium with 200 ng/mL AbA. AD-prey/promoter-AbAi was used as a negative control. **C-E**, *HuMYB132* could bind to promoters of *HuADH1* (C), *HuCYP76AD1–1* (D), and *HuDODA1* (E) using an EMSA assay. The MRE and MBS sites are marked with red letters in the sequence of biotin and mutant probes

According to PlantCARE database (http://bioinformatics.Psb.ugent.be/webtools/ plantcare/html/), all promoters of *HuADH1*, *HuCYP76AD1–1,* and *HuDODA1* have MYB cis-elements, including MRE (CAACCA and ACCTAA) and MYB-binding sites (MBS, T/CAACTG). To elucidate whether *HuMYB132* directly targets the promoters of *HuADH1*, *HuCYP76AD1–1*, and *HuDODA1* genes, their promoter regions were cloned into a pABAi vector and introduced into yeast cells. As shown in Fig. 4A, the bait yeast cells respectively harboring *HuADH1*, *HuCYP76AD1–1*, and *HuDODA1* promoter plasmids could not grow on the SD/−Ura medium supplemented with 200 ng/mL AbA. Subsequently, the full-length of *HuMYB132* was inserted into the pGADT7 vector and transformed into the bait yeast cells. Yeast cells co-transformed with *HuMYB132* and *HuADH1* or *HuCYP76AD1–1* or *HuDODA1* grew well on the SD/−Leu medium with 200 ng/mL AbA, indicating that *HuMYB132* could directly bind to the promoters of *HuADH1*, *HuCYP76AD1–1* and *HuDODA1* (Fig. 4B). Besides, an in vitro EMSA assay was performed to verify the binding ability of *HuMYB132* to *HuADH1*, *HuCYP76AD1–1*, and *HuDODA1* promoters. Clear mobility shifts in bands were detected when DNA fragments from *HuADH1*, *HuCYP76AD1–1*, and *HuDODA1* promoters containing MRE and MBS elements were biotin-labelled and incubated with purified recombinant HuMYB132 protein (Fig. 4C-E; Additional file 1: Fig. S4-S6). In addition, the shift bands became weaker with the addition of the unlabeled probes compared to no significant change happened with the presence of the mutant probes. These results indicated that *HuMYB132* can directly bind to promoters of *HuADH1*, *HuCYP76AD1–1*, and *HuDODA1* via the MRE and MBS elements.

**Fig. 5:**
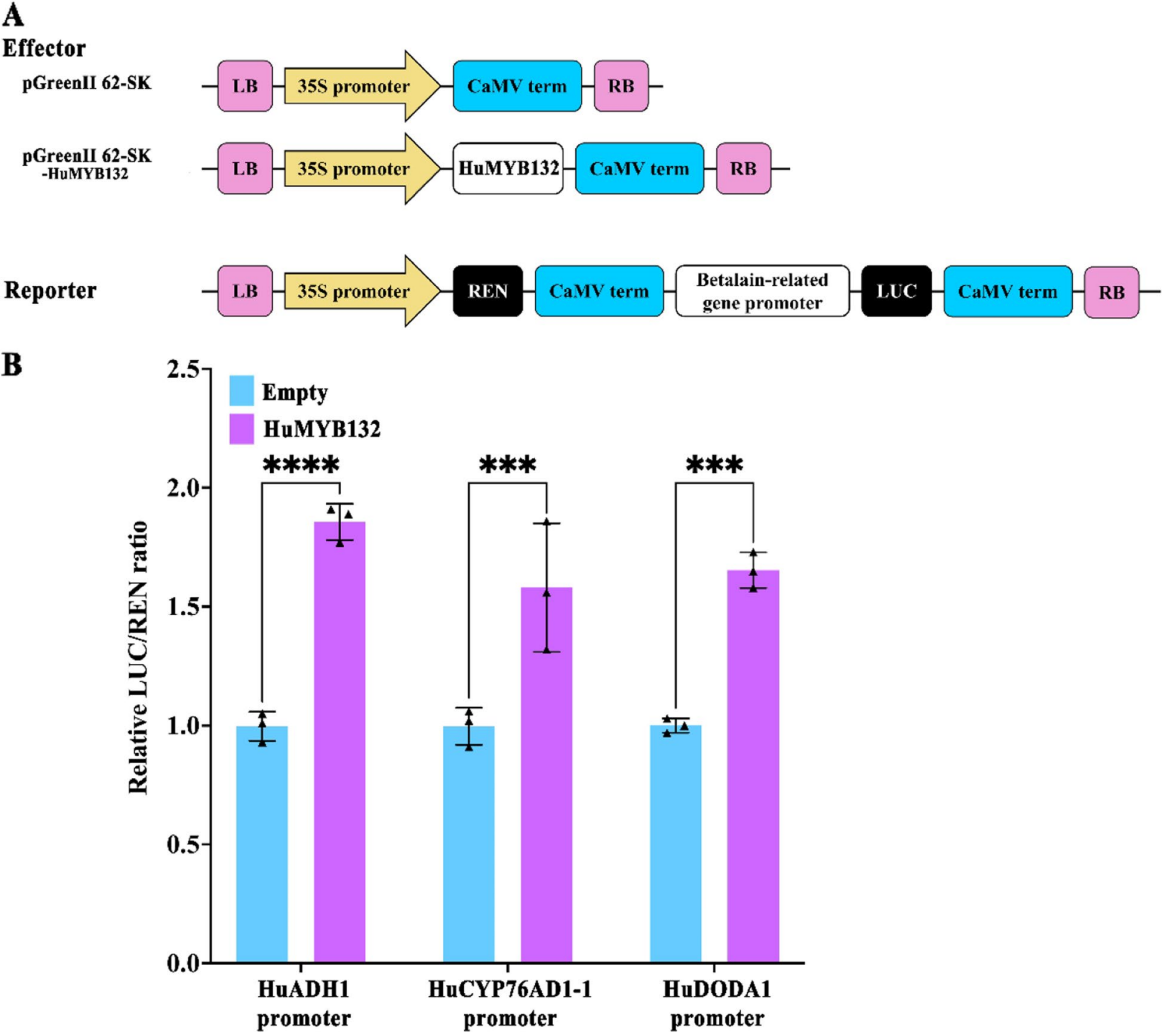
Transcription effects of *HuMYB132* on *HuADH1*, *HuCYP76AD1–1*, and *HuDODA1* promoters by dual luciferase assay. **A**, Effector and reporter vector diagrams. **B**, *HuMYB132* activated the transcription of *HuADH1*, *HuCYP76AD1–1*, and *HuDODA1* promoters by dual-luciferase transient expression assay in *N. benthamiana* leaves. The LUC/REN ratio of the empty vector (pGreenII 62-SK) plus the *HuADH1*, *HuCYP76AD1–1,* and *HuDODA1* promoters were used as a calibrator (set as 1). Small triangles represent the distribution of data for each biological replicate. Results were expressed as means ± SD (n = 3), with significance values presented as: *** p < 0.001; **** p < 0.0001

To verify the effects of *HuMYB132* on the transcription of betalain biosynthetic genes, the coding region of *HuMYB132* and the promoter regions of *HuADH1*, *HuCYP76AD1–1*, and *HuDODA1* were inserted into the dual luciferase assay system (Fig. 5A). Compared with the empty control, the infiltration of *HuMYB132* could activate the *HuADH1*, *HuCYP76AD1–1*, and *HuDODA1* promoters and their LUC/REN ratio values were significantly increased (Fig. 5B). These results indicated that *HuMYB132* could activate the transcriptions of *HuADH1*, *HuCYP76AD1–1*, and *HuDODA1* responsible for pitaya betalain biosynthesis.

### Silencing *HuMYB132* results in reduction of Betalain production

**Fig. 6:**
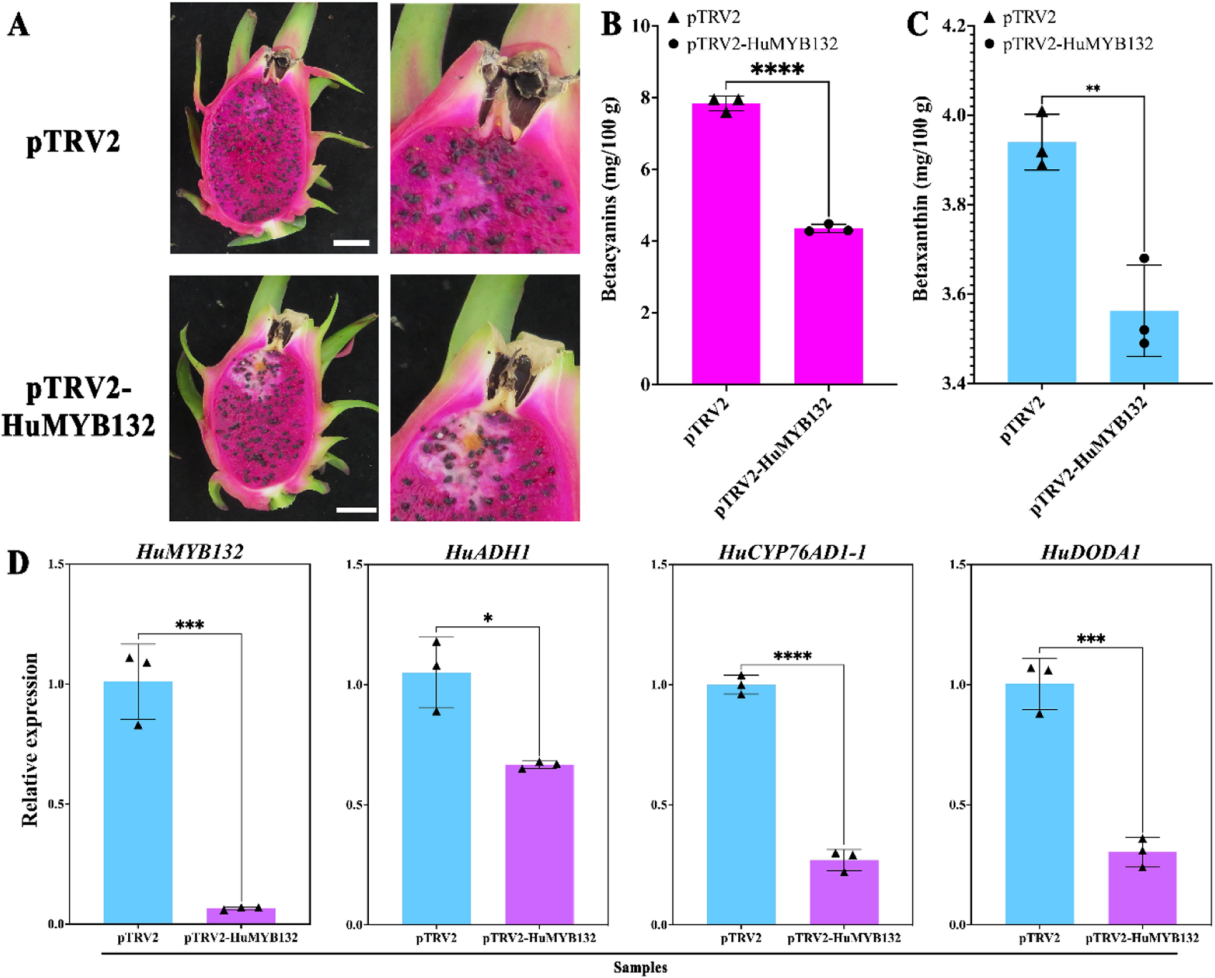
Silencing *HuMYB132* in ‘Guanhuahong’ pitaya. **A**, Fruit images after pTRV2 or pTRV2-HuMYB132 injection. Bars = 2 cm. **B-C**, Betacyanin (B) and betaxanthin (C) contents after injection. **D**, Expression analyses of *HuMYB132* in injected pitaya pulps. Small triangles represent the distribution of data for each biological replicate. Results were expressed as means ± SD (n = 3), with significance values presented as: * p < 0.05; ** p < 0.01; *** p < 0.001; **** p < 0.0001; while non-significant (ns) (p > 0.05)

A gene silencing assay was performed to further explore the function of *HuMYB132* in the pulp of ‘Guanhuahong’ pitaya. Compared with the empty vector, silencing *HuMYB132* repressed the process of pulp coloration due to the reduction of betalains, especially for betacyanins (Fig. 6A-C). Results from RT-qPCR showed that silencing *HuMYB132* resulted in significant decreases in expression levels of *HuADH1*, *HuCYP76AD1–1,* and *HuDODA1* (Fig. 6D). These results suggested that *HuMYB132* acts as a positive regulator involved in the betalain biosynthesis of pitaya.

## Discussion

**Fig. 7:**
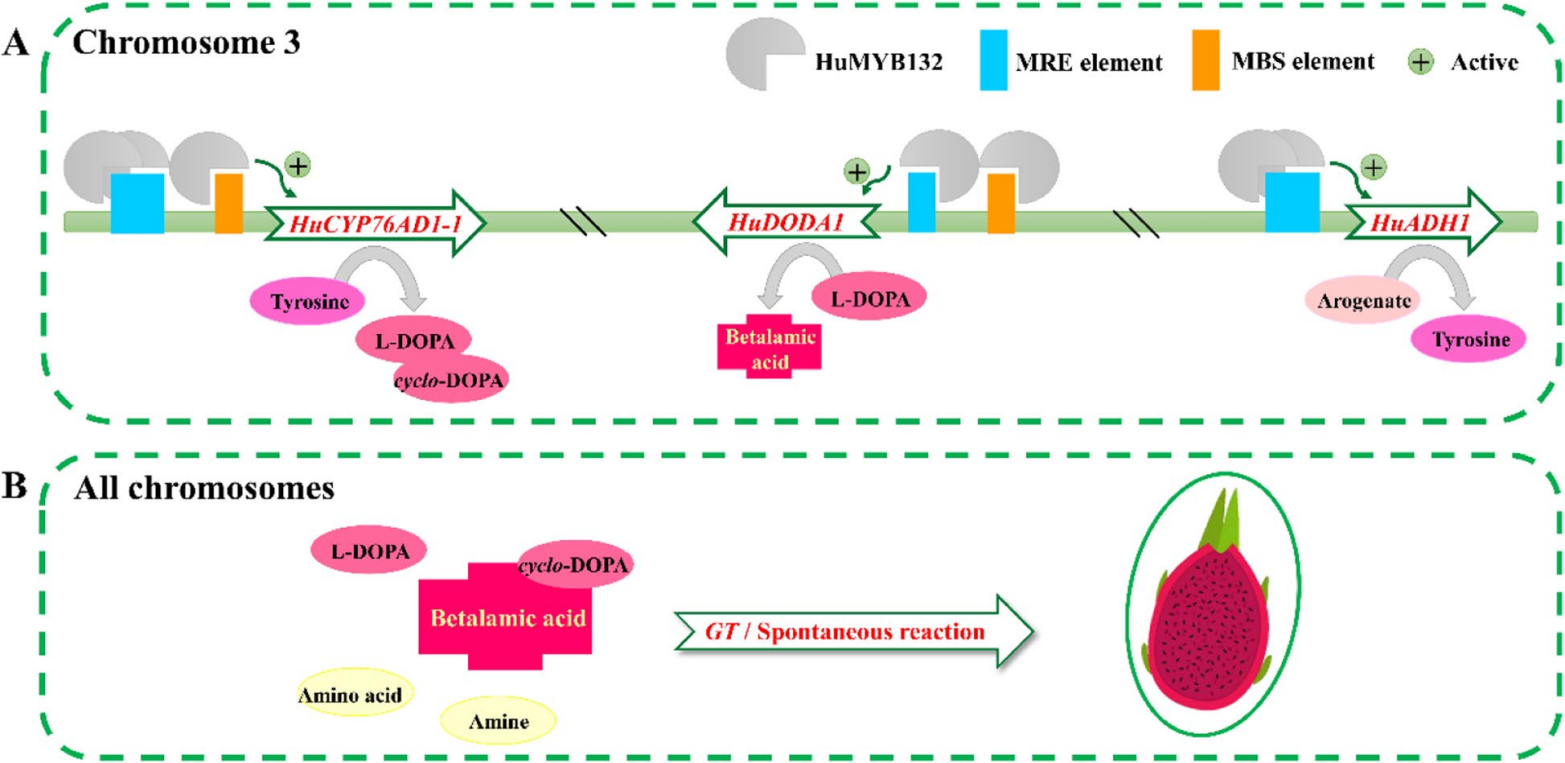
Schematic illustration of *HuMYB132* involved in pitaya betalain biosynthesis. **A**, *HuMYB132* activates the transcription of *HuADH1*, *HuCYP76AD1–1*, and *HuDODA1* by binding to the MRE and MBS elements in their promoters on pitaya chromosome 3. **B**, Betalains are synthesized in pitaya all chromosomes by *GT* genes and/or spontaneous reaction

ADH, an enzyme responsible for tyrosine synthesis, underwent a gene duplication event that resulted in the deregulated ADHα clade and the canonical tyrosine-sensitive ADHβ clade [25]. Due to gene duplication events, CYP76AD1, a novel cytochrome P450 (Cyt P450), is divided into betacyanin-functional CYP76AD1-α, betaxanthin-functional CYP76AD1-β, and unknown-functional CYP76AD1-γ [26]. DODA is an enzyme catalyzing betalamic acid synthesis, which is classified into functional DODAα and nonfunctional DODAβ based on gene duplication events [27]. In *B. vulgaris*, *C. quinoa*, *Amaranthus tricolor*, *Mirabilis jalapa*, *Stegnosperma halimifolium*, *Carnegiea gigantea* and *Mesembryanthemum crystallinum*, the functions of CYP76AD1-α and DODAα clade homologous genes have been figured out [17, 28–31]. However, the involvement of *ADHα* in betalain biosynthesis has only been investigated in beet [10]. Our previous study suggested that *HuADH1*, *HuCYP76AD1–1*, and *HuDODA1* are homologs of betalain-specific *ADHα*, *CYP76AD1-*α, and *DODA*α clades, respectively [1]. In this study, we found that *HuCYP76AD1–1* and *HuDODA1* were key structural genes, while *HuADH1* encoded a limitation enzyme in pitaya betalain biosynthesis (Fig. 1). These betalain biosynthesis genes were co-localized on chromosome 3, while *HuCYP76AD1–1* and *HuDODA1* showed a closer distance than *HuADH1* in *H. undatus*, probably implicating a more efficient betalain biosynthesis in pitaya (Fig. 7). *B. vulgaris* also has betalain-specific *CYP76AD1-*α and *DODA*α homologs on chromosome 2 that is close to each other [26]. So, *HuADH1*, *HuCYP76AD1–1*, and *HuDODA1* are three important structural genes contributing to betalain biosynthesis in pitaya.

Based on the WGCNA of betalain biosynthesis genes and potential TFs, *HuMYB132* was preferentially expressed in red-pulp pitaya at the mature stage [1], which is further verified by RT-qPCR analyses (Fig. 2). MYB-related subfamily members contain a single or a partial MYB repeat and are classified into five subgroups: CIRCADIAN CLOCK ASSOCIATED 1 (CCA1)-like, CAPRICE (CPC)-like, TELOMERIC DNA-BINDING PROTEIN (TBP)-like, I-box-binding-like and R-R-type. A root tree of *HuMYB132* and 64 Arabidopsis 1R-MYB proteins was constructed using *AT1G09770.1* (*AtCDC5*, an ‘unusual’ *MYB* gene), suggesting that HuMYB132 was a putative R-R-type MYB-like TF (Fig. 2B). The sequence analysis indicated that HuMYB132 has two separated DBD domains and a histidine region (Fig. 2C). The SHAQKYF amino acid signature motif in DBD II is highly conserved in MYB-related genes and can be used to distinguish between R2R3-MYB genes [32]. Moreover, HuMYB132 is a nucleus protein with transcriptional activator activity, consistent with the previous report that DBD I was crucial for transcriptional activation activity (Fig. 3) [24]. MYB TFs can regulate downstream gene expression by interacting with MYB-binding sites in promoters [33–35]. In our study, *HuMYB132* promoted the expressions of *HuADH1*, *HuCYP76AD1–1*, and *HuDODA1* by specifically binding to the MER and MBS elements of their promoters on pitaya chromosome 3, enhancing the reaction from arogenate to products of L-DOPA, cyclo-DOPA, and betalamic acid (Fig. 7A). Subsequently, those products were transferred into kinds of betacyanins via *GT* genes, which were located in other pitaya chromosome, and/or generate betaxanthins through spontaneous reactions (Fig. 7B). Thus, *HuMYB132* is a positive regulator and has a transcription effect on betacyanin biosynthesis in pitaya. Similarly, *HpWRKY44* and *HmoWRKY40* were also positive regulators of pitaya betacyanin biosynthesis by promoting the expression of *HuCYP76AD1–1* [36, 37]. However, more studies are necessary to determine whether *HuMYB132* can coordinate with the other TFs in regulation of pitaya betalain biosynthesis. Further studies are necessary to explore the relationships between HuMYB132 or other TFs and other key betalain biosynthesis genes.

A previous study reported that a rice R-R-type MYB TF, *MID1* (*Os05g37060*), was responsive to drought stresses during reproductive development by improving the contents of proline, soluble sugar, and the activities of peroxidase and superoxide dismutase [38]. During seed germination of Arabidopsis, *AtDIV2* (*At5g04760*, a R-R-type MYB TF) integrated ABA signaling to negatively regulate salt stress [39]. *At5g58900* (A Arabidopsis R-R MYB TF) was involved in the NO signaling pathway through binding to the *NAC027* promoter, resulting in a response to aluminum stress [40]. Moreover, *AtMYBL* (*At1g49010*) was reported to promote leaf senescence via decreasing chlorophyll content and regulating ABA or salinity signal transduction [24]. These results suggested that R-R-type MYB TFs are responsive to abiotic stresses. In general, betalain-producing plants can adapt to regions with high temperatures, drought, and salty environmental conditions due to betalain metabolites playing important roles in plant abiotic and biotic stress resistance [41]. However, more research is needed to determine whether *HuMYB132* is responsible for betalain biosynthesis in response to stress in the environment.

## Conclusion

In summary, *HuADH1*, *HuCYP76AD1–1*, and *HuDODA1* genes were critical for pitaya betalain biosynthesis. *HuMYB132* is a member of the R-R-type sub-family and it is a nuclear protein with transcriptional activation activity. *HuMYB132* positively regulates the expression of betalain biosynthesis genes by binding to the MRE and MBS elements in the promoters of *HuADH1*, *HuCYP76AD1–1*, and *HuDODA1*. Silencing *HuMYB132* reduced the expression levels of *HuADH1*, *HuCYP76AD1–1*, and *HuDODA1*, leading to the reduction of betalain content in red pitaya pulp. The present studies provide new insights into understanding the molecular mechanism of betalain biosynthesis in pitayas.

## Methods

### Plant materials

‘Guanhuahong’ (red peel with red pulp, *H. monacanthus*) and *Nicotiana benthamiana* were used as plant materials. *N. benthamiana* was grown in a greenhouse with a condition of 16 h/8 h day/night at 25 °C and was used for transient expression, subcellular localization, and dual luciferase reporter assay. The South China Agricultural University provided all plant materials used in this study, and no specific permissions were required for the collection of those samples for research purposes following institutional, national, and international guidelines. Fruits of ‘Guanhuahong’ pitaya were cultivated in the orchard of Jinsuinong (Zhongluotan Village, Guangzhou City, Guangdong Province, China) and collected on the 14^th^, 17^th^, 19^th^, 23^rd^, 25^th^, 27^th^, and 32^nd^ day after flowering (DAF). All samples were frozen in liquid nitrogen immediately, and stored at − 80 °C until future analyses.

### Expression and sequence analyses

Total RNA of infiltration *N. benthamiana* leaves, pitaya pulps (14^th^, 17^th^, 19^th^, 23^rd^, 25^th^, 27^th^, and 32^nd^ DAF), and injection pitaya pulps were isolated using the EASYspin Plus Complex Plant RNA Kit (RN53) (Aidlab Biotechnology, Beijing, China), and then cDNA was synthesized using the PrimeScript™ RT Reagent Kit with gDNA Eraser (TaKaRa, Shiga, Japan).

Reverse transcription PCR (RT-PCR) was conducted using I-5™ 2 × High-Fidelity Master Mix (MCLAB, USA) and pictured by Gel Doc™ XR+ Imaging System (Bio-Rad, USA). Real-time quantitative PCR (RT-qPCR) was performed on an CFX384-Real-Time System (C1000 Touch Thermal Cycler, USA) using the RealUniversal Color PreMix (SYBR Green) (TIANGEN, China) and specific primers (Additional file 2). The *Actin* genes of pitaya and *N. benthamiana* are respectively referred to Chen et al. [42] and Zhao et al. [43]. All determinations were performed in three biological repetitions with three technical replicates.

The full-length coding sequence of *HuADH1*, *HuCYP76AD1–1*, *HuDODA1* and *HuMYB132* were cloned from ‘Guanhuahong’ pitaya pulps using I-5™ 2 × High-Fidelity Master Mix (MCLAB, USA) with specific primers (Additional file 2). Besides, the phylogenetic tree of HuMYB132 and Arabidopsis 1R-MYB proteins was constructed by the Maximum likelihood method (ML) in MEGA 7 with 1000 bootstrap and displayed by EVOLVIEW online tool (https://www.evolgenius.info/evolview/). The protein sequences of HuMYB132, XP_021748976.1 (*Chenopodium quinoa*), XP_010673224.1 (*Beta vulgaris*), XP_021854462.1 (*Spinacia oleracea*), AT1G49010.1 (*Arabidopsis thaliana*), and RVX14086.1 (*Vitis vinifera*) were aligned by Clustal X software and shown by GeneDoc software.

### Measurement of Betalain contents

Betalains were extracted and measured following our previously described method [23]. In brief, 0.5 g of freeze-dried samples were ground into fine powder and extracted with 5 mL 80% aqueous methanol (v/v) solution by sonication for 10 min and stirred for 20 min in the dark at room temperature. After centrifuging at 5000 rpm for 15 min, the residues were subjected to a similar second extraction. The supernatants were measured by spectrophotometry (Infinite M200, Tecan Co.) at 478 nm for betaxanthins and 538 nm for betacyanins. All determinations were performed in three biological repetitions with three technical replicates.

### The transient expression of Betalain pathway genes in *N. benthamiana*

The coding sequences of betalain biosynthesis genes were inserted into the pEAQ vector to create pEAQ-HuADH1, pEAQ-HuCYP76AD1–1 and pEAQ-HuDODA1 fusion constructs (primers are listed in Additional file 2), which were separately transformed into *Agrobacterium tumefaciens* strain GV3101 and then infiltrated into *N*. *benthamiana* leaves [44]. 5 days after infiltration, *N*. *benthamiana* leaves were frozen in liquid nitrogen immediately and stored at − 80 °C for betalain content measurement and gene expression analyses. The experiments were repeated three biological replicates.

### Subcellular localization

The full-length coding sequence of *HuMYB132* was inserted into the pGreen-35S-GFP vector (primers are listed in Additional file 2), transformed into *A. tumefaciens* strain GV3101 (pSoup-p19), and infiltrated into *N*. *benthamiana* leaves. 2 days after infiltration, leaf protoplasts were isolated according to [44] and the GFP fluorescence was observed by a fluorescence microscope (ZEISS LCM-800, Germany).

### Transactivation activity in yeast cells and *N*. *benthamiana*

The coding sequence of *HuMYB132* was fused with the GAL4 DNA-binding domain (DBD) in the pGBKT7 vector. Then, pGBKT7-HuMYB132, negative control (pGBKT7) and positive control (pGBKT7-P53 and pGADT7-T) were separately transformed into yeast cells and cultured on the synthetic dropout medium without tryptophan (SD/−Trp) or without tryptophan, histidine, and adenine (SD/−Trp -His -Ade). The transactivation activity of HuMYB132 in yeast cells was assessed based on their growth status in SD/−Trp -His -Ade medium after 3 d in 30 °C, followed by x-α-galactosidase (X-ɑ-Gal) incubation for 30 min.

The full-length of *HuMYB132* was fused with GAL4BD in the BD-62-SK vector driven by the Cauliflower Mosaic Virus (CaMV) 35S promoter. Then, the effector of BD-62-SK-HuMYB132, internal control (BD-62-SK), positive control (BD-62-SK-VP16), and reporter plasmids were separately introduced into the *A. tumefaciens* strain GV3101 (pSoup), followed by the co-infiltration of effector and reporter into *N*. *benthamiana* leaves. The transactivation activity of *HuMYB132* in *N*. *benthamiana* was assessed using the Dual-Luciferase Reporter Assay System (Promega, USA) after 3 d. The experiments were repeated three biological replicates.

### Yeast one-hybrid assay

‘Guanhuahong’ pitaya DNA was isolated using Plant DNA Extraction Kit (DN14) (Aidlab, China) and Ribonuclease A (RNase A) (TaKaRa, Shiga, Japan). The DNA fragments of *HuADH1*, *HuCYP76AD1–1*, and *HuDODA1* promoters were cloned using Seq Amp DNA Polymerase (TaKaRa, Shiga, Japan) and inserted into the pABAi vector with specific primers (Additional file 2). Then, the recombination plasmids of pABAi-HuADH1, pABAi-HuCYP76AD1–1, and pABAi-HuDODA1 were transformed into yeast cells and cultured on the SD medium lacking Ura with or without 200 ng/mL AbA. After 5 days of culture on a 30 °C incubator, the yeast strains harboring pABAi-HuADH1, pABAi-HuCYP76AD1–1, and pABAi-HuDODA1 plasmid sequences were verified via Seq Amp DNA Polymerase (TaKaRa, Shiga, Japan). Besides, the cDNA sequence of *HuMYB132* was inserted into the pGADT7 vector with specific primers (Additional file 2). Then, the plasmid of pGADT7-HuMYB132 was transformed into the yeast strains containing pABAi-HuADH1, pABAi-HuCYP76AD1–1, and pABAi-HuDODA1 and cultured on the SD medium lacking Leu with or without 200 ng/mL AbA. After 5 days of culture on a 30 °C incubator, the abilities of *HuMYB132* to bind *HuADH1*, *HuCYP76AD1–1* and *HuDODA1* promoters were evaluated by the growing status on SD/−Leu.

### Dual luciferase reporter assay

The full-length of *HuMYB132* was inserted into the effector vector while the DNA sequences of *HuADH1*, *HuCYP76AD1–1*, and *HuDODA1* promoters were inserted into the reporter vector (Additional file 2). After individually transforming them into the *A. tumefaciens* strain GV3101 (pSoup), the effector and reporter were infiltrated together into *N*. *benthamiana* leaves. The ratio of LUC to Ren activity was measured using the Dual-Luciferase Reporter Assay System (Promega, USA) after 3 d. The experiments were repeated three biological replicates.

### Protein purification and electrophoretic mobility shift assay

The coding region of *HuMYB132* was fused with GST in the pGEX-4 T-1 vector and expressed in *Escherichia coli* strain BM Rosetta (DE3) (Additional file 2). The GST-HuMYB132 protein was further purified using Glutathione-Superflow Resin (Yeasen, China) (Additional file 1: Fig. S2). Oligonucleotide probes were synthesized and labelled with biotin at their 3′-ends (Sangon Biotech, China) (Additional file 1: Fig. S3). Electrophoretic mobility shift assay (EMSA) was conducted using the LightShift Chemiluminescent EMSA Kit (Thermo Scientific, USA). 50 ng GST-HuMYB132 protein was incubated with biotin-labeled probes with the addition of biotin-unlabeled probes, or mutant probes, or nothing. The shift bands of free and protein-DNA complexes were separated using a 6% native polyacrylamide gel. Then, the blots were cut after hybridisation with antibodies and transferred to a 5 × 9 cm nylon membrane. The blots were detected using the Chemiluminescent Biotin-labeled Nucleic Acid Detection Kit (Beyotime, China) and pictured by the ChemiDoc MP imaging system (Bio-Rad, USA) with the chemiluminescence method. Probe sequences used for EMSA assay are listed in Additional file 2.

### Gene silencing assay of *HuMYB132* in pulps of ‘Guanhuahong’ pitaya

The coding region of *HuMYB132* was inserted into the pTRV2 vector and transformed into *A. tumefaciens* strain GV3101 (Additional file 2). The pTRV2-HuMYB132 and pTRV1 were co-infiltrated in the pulps of ‘Guanhuahong’ pitaya and the empty vector was used as a negative control according to the method of Xie et al. [23]. Each pulp was injected with 1 mL of *A. tumefaciens*. 14 days after infiltration, pulps were frozen in liquid nitrogen immediately and stored at − 80 °C for betalain content measurement and gene expression analyses. The experiments were repeated three biological replicates.

## Supplementary Information

**Additional file 1 **The original images of RT-PCR and EMSA assays. Fig. S1. Full-length gel of Fig. 1B. Fig. S2. The SDS-PAGE gel stained with coomassie brilliant blue, presenting affinity purification of the GST (A) amd GST-HuMYB132 (B) proteins. Fig. S3. The unprocessed blots of the biotin probe of *HuADH1*, *HuCYP76AD1–1* and *HuDODA1* promoters. Fig. S4-S6. The original images of *HuMYB132* binding to the *HuADH1* (Fig. S4), *HuCYP76AD1–1* (Fig. S5) and *HuDODA1* (Fig. S6) promoters in EMSA assay.

**Additional file 2.** The primers used in this study.

**Additional file 3 **Coding sequences of *HuADH1*, *HuCYP76AD1–1*, *HuDODA1* and *HuMYB132*, and promoter sequences of *HuADH1*, *HuCYP76AD1–1* and *HuDODA1*.

## Abbreviations

ADH: arogenate dehydrogenase
ATs: acyltransferase
CYP76AD1: cytochrome P450 enzyme
DBD: DNA binding domains
DODA: 4,5-DOPA extradiol dioxygenase
GT: glucosyltransferase
EMSA: electrophoretic mobility shift assay
MBS: MYB-binding site
TFs: transcription factors
WGCNA: weighted correlation network analysis
