# Supplementary material for: Betalain biosynthesis in red pulp pitaya is regulated via HuMYB132: a R-R type MYB transcription factor
Source: BMC Plant Biol. 2023 Jan 13;23:28. doi: 10.1186/s12870-023-04049-6 (PMC9837905; doi:10.1186/s12870-023-04049-6)
Supplement: Supplementary file 1 — Additional file 1 The original images of RT-PCR and EMSA assays. Fig. S1. Full-length gel of Fig. 1B. Fig. S2. The SDS-PAGE gel stained with coomassie brilliant blue, presenting affinity purification of the GST (A) amd GST-HuMYB132 (B) proteins. Fig. S3. The unprocessed blots of the biotin probe of HuADH1, HuCYP76AD1–1 and HuDODA1 promoters. Fig. S4-S6. The original images of HuMYB132 binding to the HuADH1 (Fig. S4), HuCYP76AD1–1 (Fig. S5) and HuDODA1 (Fig. S6) promoters in EMSA assay. [file 12870_2023_4049_MOESM1_ESM.docx]

**Additional file 1.** The original images of RT-PCR and EMSA assays.

| 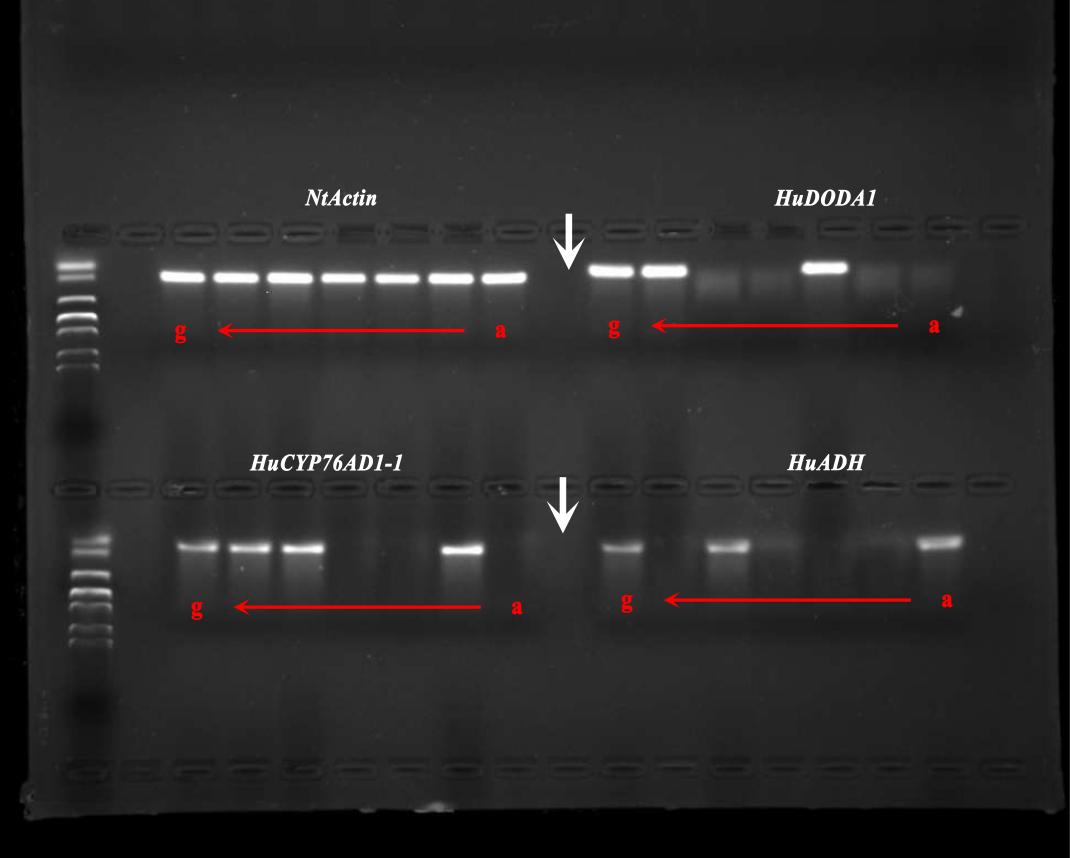 |
| --- |
| **Figure S1.** Full-length gel of **Figure 1B.** |

| 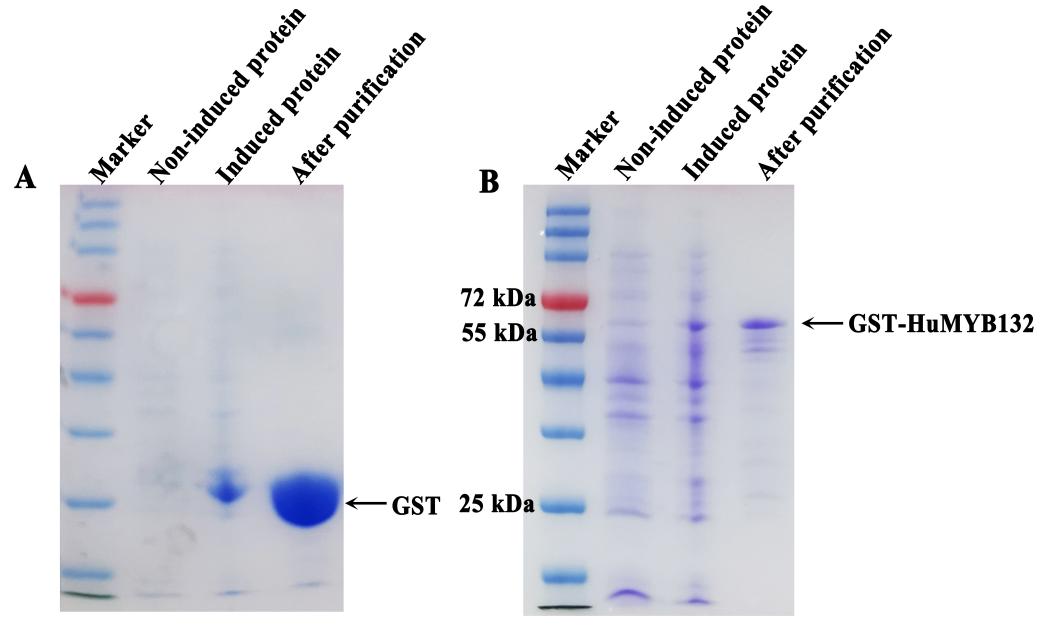 |
| --- |
| **Figure S2.** The SDS-PAGE gel stained with Coomassie brilliant blue, presenting affinity purification of the GST (A) amd GST-HuMYB132 (B) proteins. The molecular size of GST and GST-HuMYB132 were respectively 26 kDa and 58 kDa. |

| 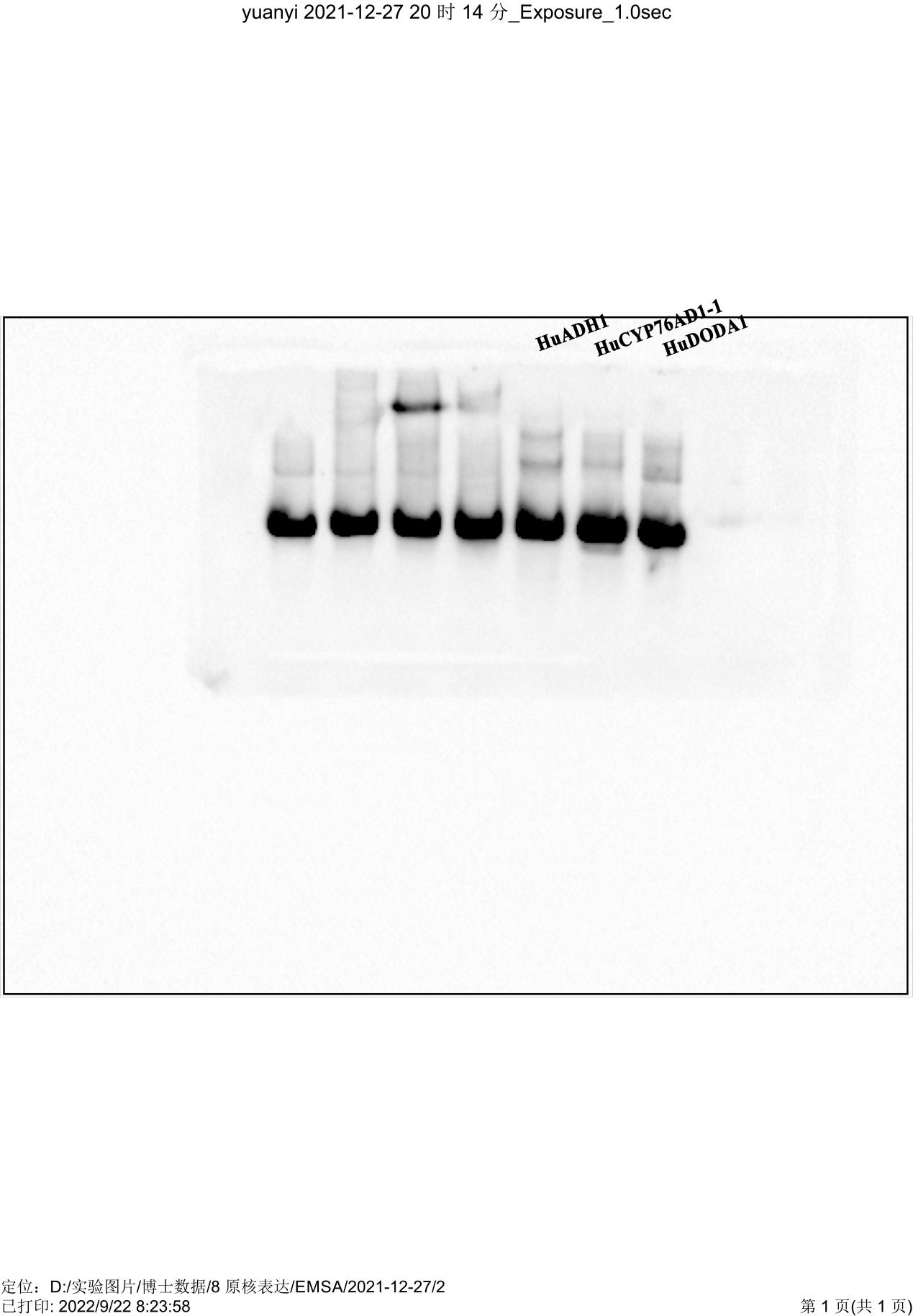 |
| --- |
| **Figure S3.** The unprocessed blots of the biotin probe of *HuADH1*, *HuCYP76AD1-1* and *HuDODA1* promoters. |

| 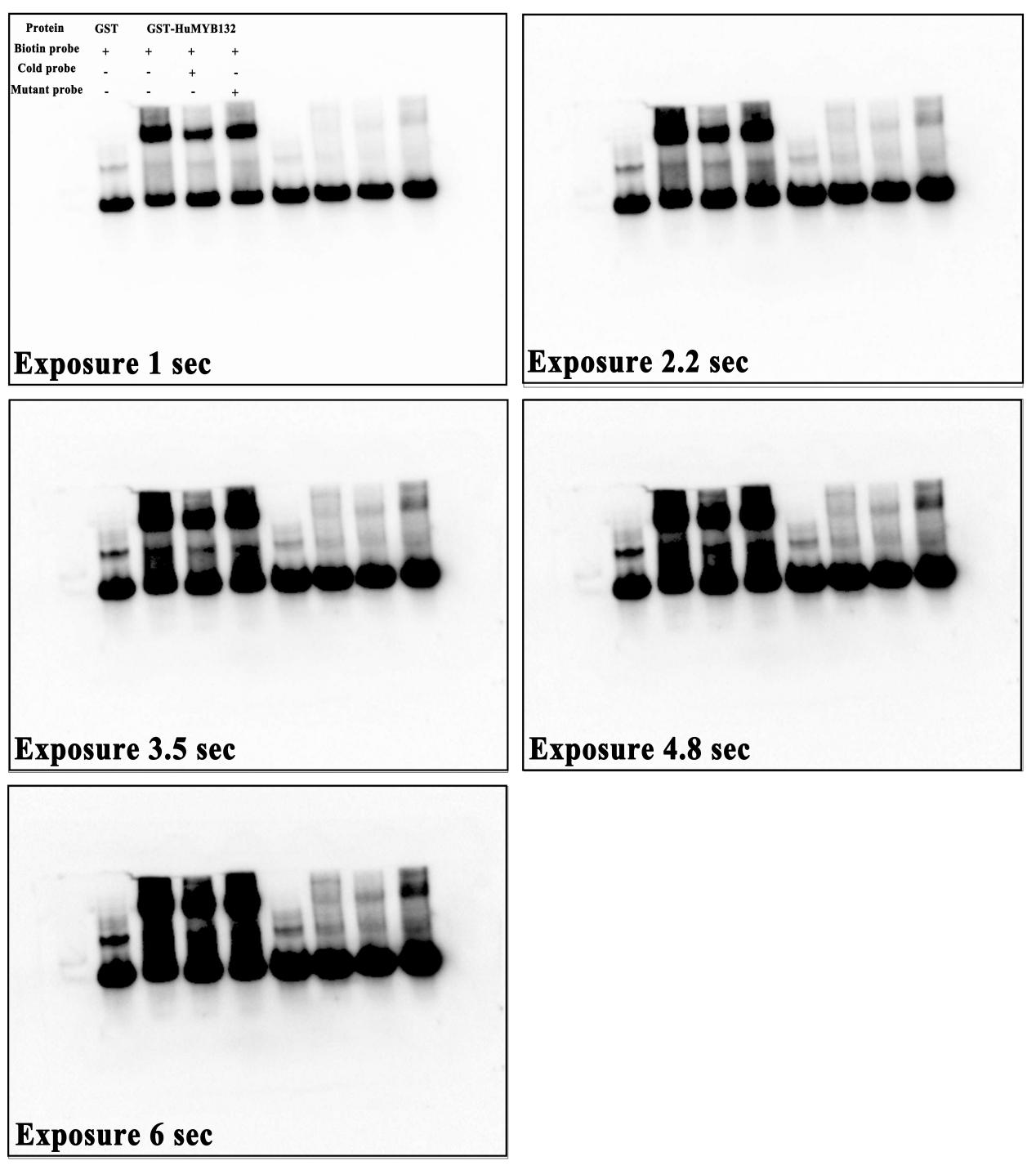 |
| --- |
| **Figure S4.** The original images of *HuMYB132* binding to the *HuADH1* promoter in EMSA assay. |

| 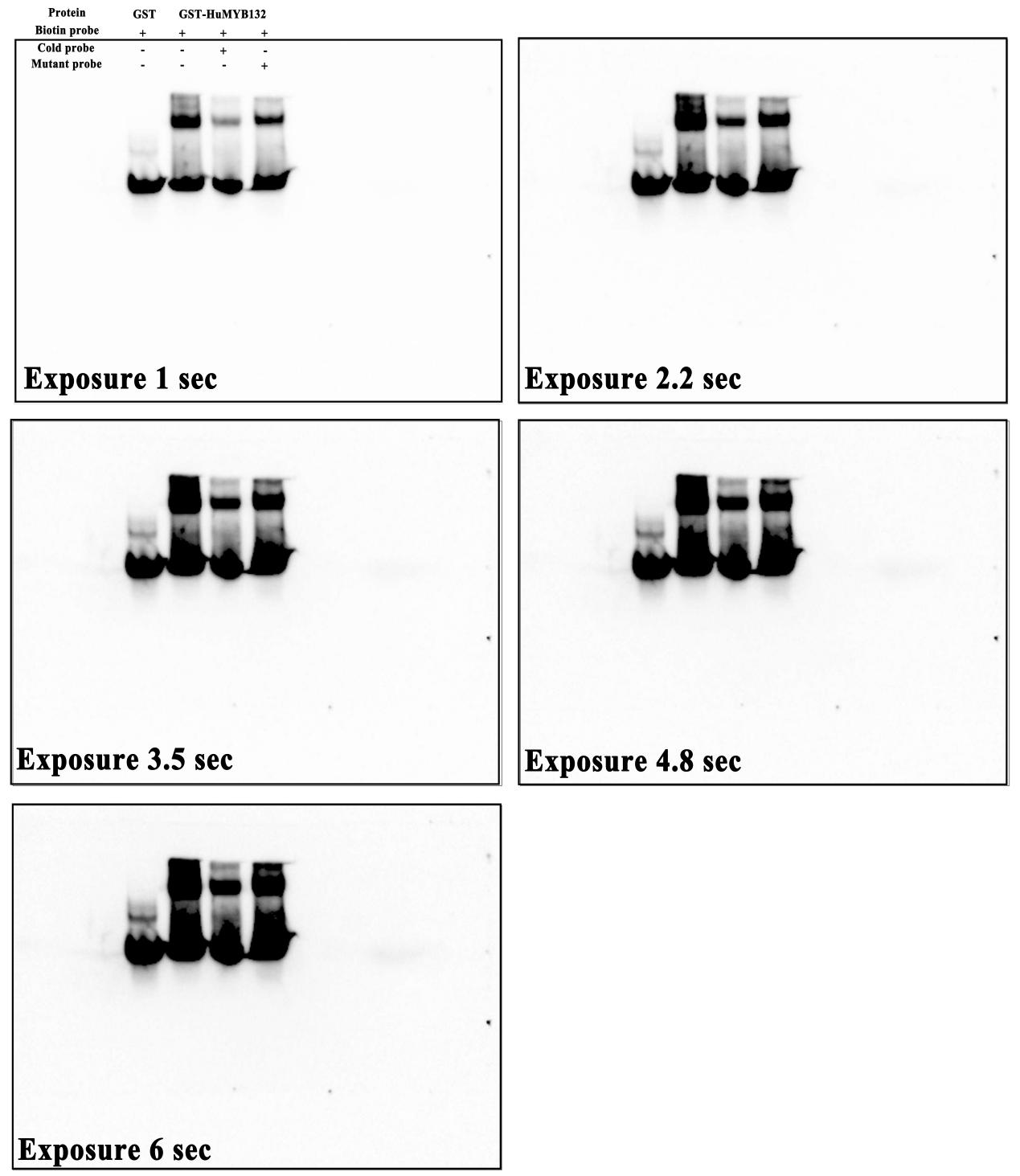 |
| --- |
| **Figure S5.** The original images of *HuMYB132* binding to the *HuCYP76AD1-1* promoter in EMSA assay. |

| 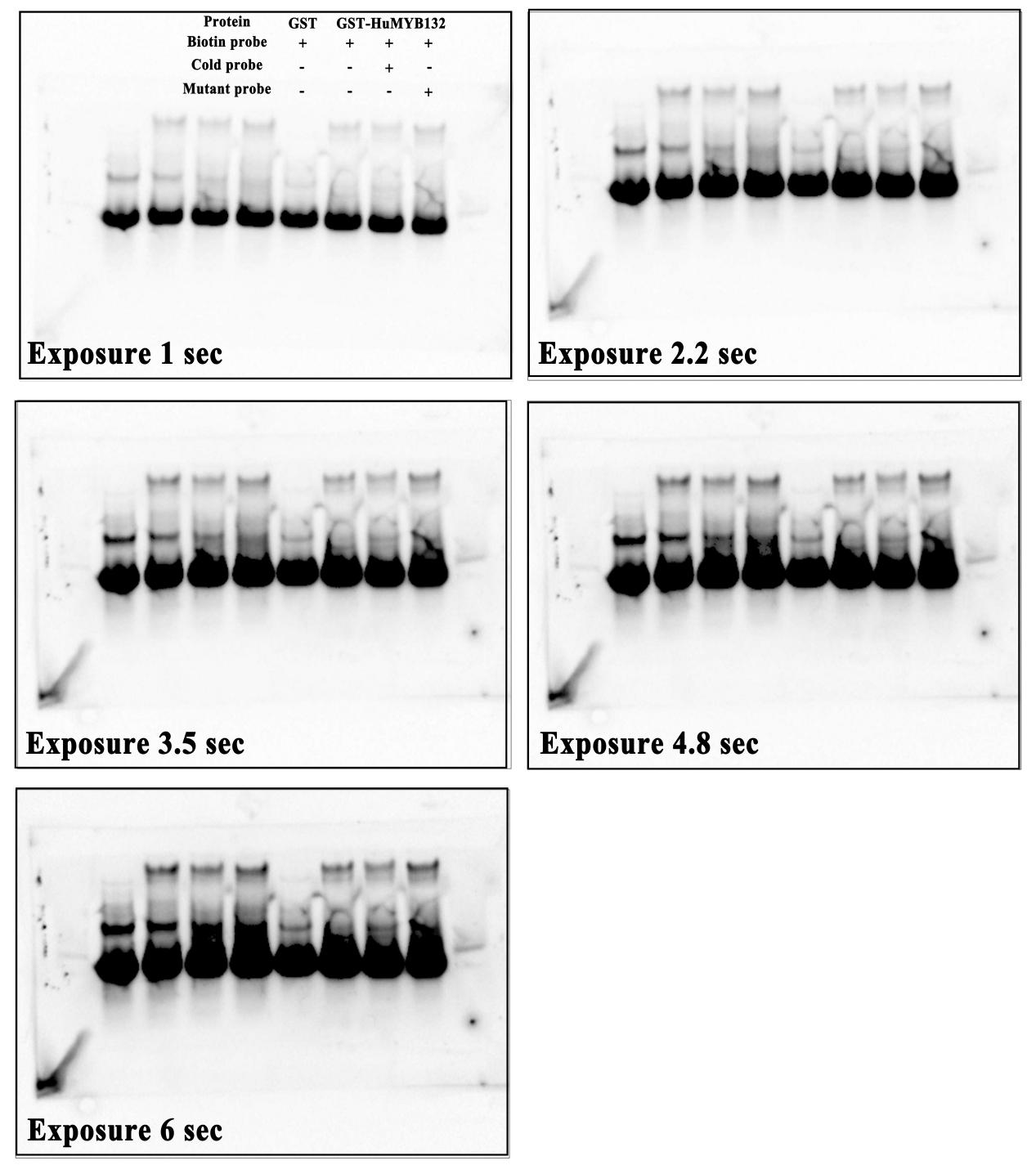 |
| --- |
| **Figure S6.** The original images of *HuMYB132* binding to the *HuDODA1* promoter in EMSA assay. |
