# Supplementary material for: Betalain biosynthesis in red pulp pitaya is regulated via HuMYB132: a R-R type MYB transcription factor
Source: BMC Plant Biol. 2023 Jan 13;23:28. doi: 10.1186/s12870-023-04049-6 (PMC9837905; doi:10.1186/s12870-023-04049-6)
Supplement: Supplementary file 2 — Additional file 2. The primers used in this study. [file 12870_2023_4049_MOESM2_ESM.docx]

**Additional file 2.** The primers used in this study.

| **Primer names** | **Primers (5'-3')** |
| --- | --- |
| **RT-qPCR assay** | |
| HuMYB132-R | TTGGAACCCCTGTGATGCTC |
| HuMYB132-F | TTGGGTAAGCCACAGGAACC |
| **CDS cloning** | |
| HuADH1-F | ATGCTATCTCTCTCCTCCACCACC |
| HuADH1-R | CTATCTCAAATTTGAGCTTCCGTTGAC |
| HuCYP76AD1-1-F | ATGGATAGCCCAACCCTCTGGCT |
| HuCYP76AD1-1-R | TCAATCCTTGCAAACGGGAATAATTTC |
| HuDODA1-F | ATGGGTGTTGGCAAAGAAGTGTCGT |
| HuDODA1-R | TCAGATGGAAGTGAACTTGTAGGAG |
| HuMYB132-F | ATGCCCAACTTGGCTTTCTGGACAAG |
| HuMYB132-R | TTATCTGTGCATTGGTGGGGGCGCCAT |
| **Promoter cloning** | |
| HuADH1-F | ATTTTCGCCCCTTTTTTTGGATTGATAAG |
| HuADH1-F | TGGTTGGGTGGAGGTTGGTGGAAATG |
| HuCYP76AD1-1-F | AATTAGTGCAGAGGTATAATACTTGCATG |
| HuCYP76AD1-1-F | GTTGGGCTATCCATGCTTTTGGGAAG |
| HuDODA1-F | ACGGCGGCGGCGATGACGATAACAACAAG |
| HuDODA1-R | TCTTTGCTTCTGGTGCTGCTGGTGCAAT |
| **Transient expression assay in *N. benthamiana*** | |
| HuADH1-pEAQ-F | ATTCTGCCCAAATTCGCGACCGGTATGCTATCTCTCTCCTCCACCACC |
| HuADH1-pEAQ-R | GAAACCAGAGTTAAAGGCCTCGAGCTATCTCAAATTTGAGCTTCCGTTGAC |
| HuCYP76AD1-1-pEAQ-F | ATTCTGCCCAAATTCGCGACCGGTATGGATAGCCCAACCCTCTGGCT |
| HuCYP76AD1-1-pEAQ-R | GAAACCAGAGTTAAAGGCCTCGAGTCAATCCTTGCAAACGGGAATAATTTC |
| HuDODA1-pEAQ-F | ATTCTGCCCAAATTCGCGACCGGTATGGGTGTTGGCAAAGAAGTGTCGT |
| HuDODA1-pEAQ-R | GAAACCAGAGTTAAAGGCCTCGAGTCAGATGGAAGTGAACTTGTAGGAG |
| **Subcellular location** | |
| HuMYB132-GFP-F | GTCGACGGTATCGATAAGCTTATGCCCAACTTGGCTTTCTGGACAAG |
| HuMYB132-GFP-R | TTTACTCATACTAGTGGATCCTCTGTGCATTGGTGGGGGCGCCAT |
| **Transactivation activity in yeast cells** | |
| HuMYB132-BD-F | CATGGAGGCCGAATTCATGCCCAACTTGGCTTTCTGGACAAG |
| HuMYB132-BD-R | GCCGCTGCAGGTCGACGTTATCTGTGCATTGGTGGGGGCGCCAT |
| **Transactivation activity in *N. benthamiana*** | |
| HuMYB132-BD -62SK-F | CGCCGTCTAGAACTAGTGGATCCATGCCCAACTTGGCTTTCTGGACAAG |
| HuMYB132-BD -62SK-R | TCGATAAGCTTGATATCGAATTCTTATCTGTGCATTGGTGGGGGCGCCAT |
| **Yeast one-hybrid assay** | |
| HuADH1-pAbAi-F | ATTGAAAAGCTTGAATTCGAGCTCGTAACCAAAGGATCGCACCACCTCCGCT |
| HuADH1-pAbAi-R | TACATACAGAGCACATGCCTCGAGTAGCTAGCTAGGAATGCGTATGCAT |
| HuCYP76AD1-1-pAbAi-F | ATTGAAAAGCTTGAATTCGAGCTCTCCATCCCCCTCCTCACGTGACCCAT |
| HuCYP76AD1-1-pAbAi-R | TACATACAGAGCACATGCCTCGAGGAGGGCGGGTTAGGTGGTTGCTGAG |
| HuDODA1-pAbAi-F | ATTGAAAAGCTTGAATTCGAGCTCGGTTAGGGGACGAATGATGCACAAAT |
| HuDODA1-pAbAi-R | TACATACAGAGCACATGCCTCGAGCGTCCGGCATGATCAGTGGTAAGGT |
| HuMYB132-pGADT7-F | GGAGGCCAGTGAATTCATGCCCAACTTGGCTTTCTGGAC |
| HuMYB132-pGADT7-R | CGAGCTCGATGGATCCTTATCTGTGCATTGGTGGGGGCGC |
| **EMSA assay** | |
| HuMYB132-GST-F | GGTTCCGCGTGGATCCATGCCCAACTTGGCTTTCTGGACAAG |
| HuMYB132-GST-R | AGTCACGATGCGGCCGCTTATCTGTGCATTGGTGGGGGCGCCAT |
| HuADH1-biotin-F | GATCGATTCTCAGCAACCACCTAACCCGCCCTCCCATTTTCCCTCTCCTC |
| HuADH1-biotin-R | GAGGAGAGGGAAAATGGGAGGGCGGGTTAGGTGGTTGCTGAGAATCGATC |
| HuCYP76AD1-1-biotin-F | ATCCATCAACCTAACCTAAATTTTATAGAATAGAATAACTGACTCAGCTC |
| HuCYP76AD1-1-biotin-R | GAGCTGAGTCAGTTATTCTATTCTATAAAATTTAGGTTAGGTTGATGGAT |
| HuDODA1-biotin-F | AAAGACTTCAGTTGAGTATAAAAGGTTAGGGGACGAATGATGCACAAATT |
| HuDODA1-biotin-R | AATTTGTGCATCATTCGTCCCCTAACCTTTTATACTCAACTGAAGTCTTT |
| HuADH1-mutation-F | GATCGATTCTCAGAAAAAAAAAAACCCGCCCTCCCATTTTCCCTCTCCTC |
| HuADH1-mutation-R | GAGGAGAGGGAAAATGGGAGGGCGGGTTTTTTTTTTTCTGAGAATCGATC |
| HuCYP76AD1-1-mutation-F | ATCCATCAAAAAAAAAAAAATTTTATAGAATAGAAAAAAAAACTCAGCTC |
| HuCYP76AD1-1-mutation-R | GAGCTGAGTTTTTTTTTCTATTCTATAAAATTTTTTTTTTTTTGATGGAT |
| HuDODA1-mutation-F | AAAGACTTAAAAAAAGTATAAAATTTTTTTGGACGAATGATGCACAAATT |
| HuDODA1-mutation-R | AATTTGTGCATCATTCGTCCAAAAAAATTTTATACTTTTTTTAAGTCTTT |
| **Dual luciferase reporter assay** | |
| HuMYB132-62SK-F | GGCCGCTCTAGAACTAGTGGATCCATGCCCAACTTGGCTTTCTGGACAAG |
| HuMYB132-62SK-R | ATCGATAAGCTTGATATCGAATTCTCTGTGCATTGGTGGGGGCGCCAT |
| HuADH-pro-0800-F | TATAGGGCGAATTGGGTACCATTTTCGCCCCTTTTTTTGGATTGATAAG |
| HuADH-pro-0800-R | TTGGCGTCTTCCATGGTGGTTGGGTGGAGGTTGGTGGAAATG |
| HuCYP76AD1-pro-0800-F | TATAGGGCGAATTGGGTACCAATTAGTGCAGAGGTATAATACTTGCATG |
| HuCYP76AD1-pro-0800-R | TTGGCGTCTTCCATGGGTTGGGCTATCCATGCTTTTGGGAAG |
| HuDODA-pro-0800-F | TATAGGGCGAATTGGGTACCACGGCGGCGGCGATGACGATAACAACAAG |
| HuDODA-pro-0800-R | TTGGCGTCTTCCATGGTCTTTGCTTCTGGTGCTGCTGGTGCAAT |
| **Gene silencing assay** | |
| HuMYB132-pTRV2-F | TCTAGAAGGCCTCCATGGGGATCCAAAACCATTGAAGAGATTAAGCTACACT |
| HuMYB132-pTRV2-R | TAATGTCTTCGGGACATGCCCGGGTAGCCTTATGAAGTACTTCTGGGCAT |
| qHuMYB132-pTRV2-F | GTGCAATTGAAGCGGGAAAAGT |
| qHuMYB132-pTRV2-R | GGAGGAATGCCCCAATCCGGAC |
