# Supplementary material for: Betalain biosynthesis in red pulp pitaya is regulated via HuMYB132: a R-R type MYB transcription factor
Source: BMC Plant Biol. 2023 Jan 13;23:28. doi: 10.1186/s12870-023-04049-6 (PMC9837905; doi:10.1186/s12870-023-04049-6)
Supplement: Supplementary file 3 — Additional file 3 Coding sequences of HuADH1, HuCYP76AD1–1, HuDODA1 and HuMYB132, and promoter sequences of HuADH1, HuCYP76AD1–1 and HuDODA1. [file 12870_2023_4049_MOESM3_ESM.docx]

**Additional file 3.** Coding sequences of *HuADH1*, *HuCYP76AD1-1*, *HuDODA1* and *HuMYB132*, and promoter sequences of *HuADH1*, *HuCYP76AD1-1* and *HuDODA1*.

**>*HuADH1***

ATGCTATCTCTCTCCTCCACCACCACCAACCCCCACTCCCGTCCTCCGCCTCACGCCCACCAACACTGCTTTCACTCTCCTCCGCCCTCTGTTCTCTCTCTCCCCAGCGCCTCCTCCCCCCGCCGCCTCCGCCTCTCTCTCCGCTGCTCCGTAAATGGGTCCGACATCCTCGCCCCCGGCGTCGGAATCTTCGCCGGAGAAGGGTTCAGTGCTGCTTCTGATGATGATGGTGAGAAGACGACGAATTATAAGATGAAGATTGCGATTGTGGGGTTCGGAAATTTCGGGCAGTTTCTTGCGAAGGAAATCGTGAAACGGGGGCACACTGTGTTGGCCTACTCTCGCTCCGACTACTCCCGCGTCGCCGCCGAGATCGGCGTTCAGTTCTTCTCCGACGCCGATGACCTCTGCGAGGAGCATCCCGAGGTGGTCCTGTTGTGCACCTCGATCCTCTCGACGGAGAAGGTCCTCCGAGGGCTGCCCCTCAATCGTCTCCGACGGTCCACCCTATTCGTGGACGTCCTGTCGGTGAAGGAGTTCCCCAGGTCACTCTTCCTACAGCTGCTCCCGCCGGACTTCGACATCCTATGCACCCACCCCATGTTCGGGCCGGACTCGGGCAAGGCCGGGTGGGGCGGTCTCCCTTTCGTCTTCGACAAGGTTCGGGTGGGGTCGGACCCGGCCCGGATCGCCCGGGTGGAGGCTTTCCTGGACGTGTTCTGCGCGGCTGGGTGCAGGATGGTGGAGATGAGCTGTGCCGAGCACGACCGGTACGCGGCCGGGTCCCAGTTCATAACCCACATGATGGGGCGGGTCCTGGAGAAGTTGGGCCTAGGGAACACCCCGATAAACACCAAAGGGTATGAGAGTTTGCTGAACTTGGTGGACAACACAGCAAGAGACAGCTTTGAGCTGTTTTATGGGCTGTTTTTGTACAACAAGAATGCAATGGAGCAGCTGGAGAGAATGGATTGGGCGTTCGAGATGGTGAAGAAGCAGCTTTCTGGGCATTTGCATGGACTTGTGAGGAAGCAGTTGATGTTGGAGACTAATTGTTCTGGGAATGACGATGTTAATGTTGGAGAGAGAGATTCAAAGCCGATGATGCTTCCTCCTCCAACGACTCCCACAAAACTTGTTCCTGAGAAACAGCAACAACTACAGCATGACTTGGTTGTCAACGGAAGCTCAAATTTGAGATAG

**>*HuCYP76AD1-1***

ATGGATAGCCCAACCCTCTGGCTTTTCATCTTTGCCTCAATCTTTTACTTCATAACTTTTCAAATTGTGAAGCTAGGGTTTAATGTGGTCATGACCTCTAAAAAAACCAAAAGAAGAAGACCCCCCTTACCTCCGGGCCCCAAGCCATTGCCCATCATAGGCAATGTGCTCGAGCTCGGGCCGAAGCCACACCGCTCGTTCGCCGACCTAGCCAAGGTCCATGGTCCACTCATGTCCCTCCGGCTAGGTAGTGTGACCACGATTATCGTGTCATCCTCTGATGTTGCCAAAGAAATGTTCCTTAAAAATGACCAACCCTTGAGCTCCAGCCGAACCATACCCAACTCAGTCACGGCTGGGGATCACCACATGCTGACCATGTCTTGGCTCCCAGTCTCCCCCAAATGGCGGAGTTTTAGGAAGATCACCACCTTCCACCTTCTCTCCCCCCAGCGCCTCGATGCTTGCTCTAGCCTTAGGCAAGCCAAGGTGCAGCAGCTATTCGAGTACGTTCTGGAATGTTCTAGAACCGGCCAGGCCGTCGATATAGGCAAGGCTGCTTTCACGACGTCCCTTAACTTGTTGTCCAAGCTGTTTTTTTCTTTAGAGTTGGCTCACCATAGATCTAGCAAGTCTCAAGAGTTTAAGGACTTAATTTGGGATATTATGGAGGATATTGGGAAGCCTAATTACGCGGATTATTTCCCATGCTTAAAGTACTTTGACCCATGTGGAATACGACGTCGTTTGGCAAATAGTTTTGAGAAATTAATTGAGGTCTTTCAAGGTATTATTCGTCAAAGGCTATCCCTGTCATCTGGCTCTCATACTCATAATGATGTGTTAGATGTTCTTCTTCAATTGTACAACCAAGAGGAACTCACCATGGACGAGATAAACCATCTGCTCGTGGATATATTTGATGCCGGAACAGACACCACTTCCAGTACATTTGAATGGGCCATGGCTGAGTTAATTAAAAATCCGACGATGATGGAGAAAGCTCAAGCTGAAATCAAAGTGGTTCTTGGGAAACAGTCGCATATTCAAGAGTCCGATATCCCAAAATTGCCTTATTTGCGGGCAATTATCAAAGAAACATTGCGTCTACACCCTCCTACTGTATTCCTCCTACCTCGTAAGGCTGAGACCGATGTGGAACTCTATGGCTACACCGTACCAAAAAATGCACAAATACTGGTGAACTTGTGGGCCTTAGGTCGAGACCCCAAAGTTTGGGAAAACCCAGAGGTGTTCTTACCTGAAAGGTTCCTGACTTGCGACATCGATGTTAAAGGAAGAGATTTTGGACTACTGCCTTTTGGGGCAGGAAGGCGAATATGTCCTGGGATGAATTTGGCGTACAGAATGCTGACCTTAATGCTCGCTACGCTTCTACAATCGTTTGATTGGAAACTCCCAAATGAGATGAACTCCAAGAATTTGGACATGGATGAAAAGTTTGGAATAGCATTGCAAAAGACTAAACCCCTTGAAATTATTCCCGTTTGCAAGGATTGA

**>*HuDODA1***

ATGGGTGTTGGCAAAGAAGTGTCGTTCAAGGAGACTTTCTATGTGTCTCATGGGAATCCGGCCATGTTGGCCGATGTGTCGTTCATAGCACGGAACTTCCTGCTGGGGTGGAAGACGAATGTCTTCCCCATCAAACCCAAGTCGATCCTGGTTGTCTCTGCTCACTGGGAGACTGATGTGCCTTCTGTATCTGCCGGTGAACATCCTGATGTCATTTACGATTTCAGCGATGTTCCTGACTGTATGTTCCAGATGAAGTACCCAGCTCTAGGGTCACCAAAACTGGCCAAAAGGGTGCAGGAGCTACTGATAGCAGGAGGGTTCAAGACAGCGAGCCTAGACGAGAGTCGTGGGTTCGACCACAGCTCATGGGTGCCCCTGAGCCTCATGTACCCTGAGGCTGACATCCCGGTGTGCCAGCTCTCAGTCCAGCCTCACCTAAGCGCGAGCCACCACTTCGACATAGGGAGGGCTTTGGCTCCTCTCAAGGAGGAAGGGGTCCTGTTCATTGGGTCTGGGGGTGCAGTGCACCCTTCTGATGACACCCCACACTGGTCTGATGGGGTTGCCCTTTGGGCTGCTGAGTTTGATCAATGGCTTGAGGATGCTCTCATTAATGGAAGGTACGATGATGTGAATAATTATCAAACAAAAGCACCTTCTGGGTGGAAAATAGCACATCCAATTCCAGAACACTTTTTACCGTTGCATGTAGCCATGGGTGCAGCTGGTGAAAAATCAAAGGCAGAGCTCATTTATCGTACGTGGGATCATGGTACTCTTGGCTATGCCTCCTACAAGTTCACTTCCATCTGA

**>*HuMYB132***

ATGCCCAACTTGGCTTTCTGGACAAGGGAGGAGGAGAAGGCGTTCGAGAACGCGATTGCTGTGCACTCAATTAGCGAACCCGATGAAGAAAAATGGGATAAGATTGCTTCTGTGGTTCCAAGTAAAACCATTGAAGAGATTAAGCTACACTTCCGAGATTTAGTTGATGATGTGGGTGCAATTGAAGCGGGAAAAGTCCCACTTCCCACTTATGGGAATGAAGAGGCTTTGTCAACTTCTAGCAAGGATCAAATGCCCTTCTCGAAGGAGCAGAAAGGAAATACCAATCAAGGAAATGGACAGTCCGGATTGGGGCATTCCTCCATGGGGAATGGAACTAAAGGAAGCTCGAAGCTCGATCACGAGCGGAAAAAAGGGATCCCTTGGACTGAGGAAGAGCACAGGTTGTTCCTACTCGGACTAGACAAATTCGGGAAGGGTGATTGGAGGAGCATATCGAGGAACTTCGTGATAACAAGGACACCAACACAAGTGGCTAGCCATGCCCAGAAGTACTTCATAAGGCTAAACTCAATGAATAGGGATAGGAGAAGATCAAGCATCCATGACATTACAAGTGTGAATGGTGGAGGTTTATCAGCAAATCAAGCGCCCATTACAGGGCAACAAAACAGTGGCACTGCACCGGCAGTTGGAGCACCAGCAGCAAATAAACATAGGGCTCAGCCCAATGTGACTCCAGGGTTAGGAATGTATGGGACCCAAATTGGTCACCCTGTTTCACCAGCAGCTCCACCCCCACCTCATTTGGGGGTGTCCGCTGTTGGAACCCCTGTGATGCTCCCCCCTGGTCATCATCACCCCCCTCTCCCACCTCACTATGCGGTTCCTGTGGCTTACCCAATGGCGCCCCCACCAATGCACAGATAA

**>*HuADH1-promoter***

ATTTTCGCCCCTTTTTTTGGATTGATAAGTGCGGGCTATATATATATTAGGCTCCGATCTTAAGAGAAGTTTTAGTGTGGGAAGAGGTCCGGGGGAACGTGGGATTAGTTTTGACCTATTGCCTTTTGCTTAGCTTGATGGAAGTTCGGGTTTGGTTGAATCGAAGGTACGGTCACCTTTTGATTTAAATTCGTATTGAAGGTCGTTCGAGGCTAACCTTGTACAATGGATTAGTGGACCAGTTACTAGCTAAAACAAAAACTAATTTTGAAAATGTGCTAGGTAAACGATTTTATATTACATTGCACATGCAATTAAGTAATTAACATTAATAAAAAAAATATAAGGACAGATTTTTGTTATCACTTTACAACAACTTCGTTCCGTGTGGGTATCATTTGCGTGAGATTTGATGAGATTCATATACGAGTCATCTTATCAATGTAAACATCTCTGTGTAGAAAGTGATTGGTTCCAAAAGTCAAAACAAAAAGATAAAATATTTTAGAATTGAAAATCTTTTAACATATAAAAAACAATACACATGGTTTGGTTGATGGGTTTGAGTTTCCCAAGAAAGATAATAACTCTTATTTTAAATTTTGGGCTATATATTATATATGACAGTCACGTCGATTGAGATAAAATTACCTACATCTGGATCTCCCCAAATTCCACAAAAGCAGAACTCACACTGGGAGGTGTTGTTGTTGTTGTGTGTGTGTGTGTGCGTGTGTGTGTGTATATATTATAGTAAGAGGAACCATAAATGTATGGAGATACGGTACCTATACATATAACAATATGTATAACATACTCTTAGGAGTATGTACTCTCACAAATCACATCATTAAATATATATTAAAAAATTAATGTATGATAAATACGCACATAAAAAATATATACACATGAACAATAAGAGTATGTACACTCGGAAGCACATAAGAAATTGACTACATATAATGCCTTTTATCTTTAGGCGTGATACACGGGATGCGAGTAGAATGGGCCATGGAGTAGAAATGTAGAATAATTAATGCTTCCAAAAATAATGTGGGGGGCTGAAATAACCATAATAAGTTATTATTAAGTTTATATTTAATATCATGAAGTATGCAATTTTACATACATATATAATTCATGAAATATTTCTCATTTAATTTTTTCAGTTGCAAGGACTCGATATTGGATCTCAATTTAAACCTGGAAACTTGAGTTTTAACCTCATTATCCCCTCATTATAAGTATTGTTGTGTATAGAACTCTATATTTTCGTAAAGTTTCTTCAAATTATAAACACAATTAACCATACAATACAAAATTTAAGTATAAACAATCTTTTACTTTTAACCAAGGAAGATAATATTTATTGTCGTGAAATCTAAGACTCCATTTGGACTTTGAAAAATTTGGTGAAAATAAAAAAAAATTGGAAAAATTACTTTTTTTTTGTATTTAGTTTGGAGAAAATTACAAAGAAAAAAAATATATTGAGGAAAGTTAGGTGAAAAAATTGTTAAAAATAAGTGATAAAATTTTCCTCATAAAATTTTGGGAAAAGTTTGAGGAAAATTTCCAACCTCCTCCAACTTCACATTACTTAGATGACACCAACATAATTCAAGCAGTTCATTAATATTCAAAATTTTCATCTAAATTCTTATATTTTTTTCTCAAATTCAAACAAGAGAAAAAAAAACTCATTTTTTTTACTTTTTTTTTATATTTTTTTCTTCAATTTTTCTTTCCATTAAATTTTTTAAGAATCAAACGGAGCCTAAAGGACTTAATATTCAAGAAGAATGCATGGTTCAGAGCAAGGGTTATTGTCTTCTCCCCCACCGTTATCTCAAGTCTCACCAACCCTCCATCCCCCTCCTCACGTGACCCATACTTTTTTTTTTTTTTTTTGGGCAACAATGACCCATACTTTTATGCTTCAGTCATTTTAATTTTCTTTAAGAGACTATTTTCAATCATTATTGTGCTGTGCACCCACCCTGAGTAATAAATATCATTTTATTACCCACCCACCACTTGTCAAAATTAAGCCTTTGAAAAAACAGAAGAAAAGCAGTCGAGAAATTCCTCTATAAATTCCATCTTTTGTTCCCTGAATGACTCGTTGCAAGATCGATTCTCAGCAACCACCTAACCCGCCCTCCCATTTTCCCTCTCCTCCCAAGTCTCCGCTTCCCACCCTTGTCTCTCTCCTCTTCTCAGGAATTAATTACACACAGCTAGCTCAGCCGCCATTTCCACCAACCTCCACCCAACCATG

**>*HuCYP76AD1-1-promoter***

AATTAGTGCAGAGGTATAATACTTGCATGTTAGATTTGGTTTCTTATTCGAGGTATCGGATAAGAACTTACCCAATCATTGAAGAAAAGTTACAATTTAAGGCCACAAGTCATAGCTTTCTTAATGAAAAAGACTACTTTTTTTTTTCCCTCAATCTATCAGTTATGGGCAAAATGTTGTTGATGTAGTGTTTGGTTGGGAGAAAAATTATTTTTCAAAAAATAATTTTTCAATCTTTCGTATTCGACATAGTAAAGGATGGAAAACTATTTTTGAATGAAAATGATTTTTCCCTCTTTTTAGGAAGATAAGAAGTTGATTTTCTTTCTTCAATTCTTTGACAATATAATCAGCTAAAATTTAGGCACAAAATTTGATTATCAACAGTTTGAATCAAAACTTGATTATCTGATGAAAAAGACTAAAATAAGCTCAAATCTAGATTTTCAACTCAAATTTGTTTTAGTTGATTGACATTTATCTCTACCATGATAATTTTATCGATATTTTTTGTCATTAAAATCATTCAGTCAACTCGCCACTAAAATAACTTTAACAATTTGGATCGAAACTTGATCATCAAATAGCAAATATTAAAATCGGCTCAAATTCAGAGTTTGAGCTCGATATTTTTCCATTTGGTCAACACCTTTTTCCACAACGACAATTCAATTGATATTTTTCACCTCTTAAATAGTGAGGTCAATATTTTTCACCACTAGAACAATTTCAACATGTTGGATTGAAACTTGATCATCCAATATGAAAGATTAAAATAGGCTCAAATTCAGATTTTGAGTACAATATTCCTTCATTTGGTCAATATTTTATCTGTTACGACAATTTTAATTGATATTTCTCGTCACTTAAACAATTAAGTTGATTTTTTTGTAACTAAAACAATTTCAACGGTTTAGATTGAAACTCGGCTATCCAGGCAGAAGTGTTCAAAACACAAATATAAATTTTAAGCTCAAAATTCCTTCGTTTGTTAAACATTTATCTCTACTATGACCATTCAGTCGATATTTTTCGTCATTTAAATAATTCAATTGGCGTTTTTTTTTTCATGATAAATGATTTATTGTGTACCAAATGACCAAAAATGTGACATAAAACTCAATTTTACTTGGAAAAACGTTTTCCACAGAAAATGATTTCTTGGAAAAAATTGTTTTCCGCAAAAAATGATTTTTCGAGAAAACAACTTCCTTTGGAACAAAACACTATCTTAATTAAAACATCTTATATGTGCTTTGGAAGGGCACGCACCACGTATATGATTTTTTATTGGACATTTCATTTGTATATTTCAATAAACTTTTTTGTAGACAACATCAATCTCTTTGTCCTTAATTATAAACCCTTAAAAATTATATTTCACATCTTTTCATTATGTCAACAAAAAAATCCTATGAGATATATAGCATTTTGCACTGAATAAAATGACCATATTTAAATGGAAAGAAAAAAATCATGTACAATTTCAAATTTCAGCATTTTTTTTTAATAATTGAAGCCAAAGGCGGGCCATGGCAGGAGCAGTAAAACAGATAACTTATTTGGCCGGCACTTGCAAGTAACCAAAGGATCGCACCACCTCCGCTGGTCTGTAAATAATATTGTCCAGCCTAAAATTGATCCATCAACCTAACCTAAATTTTATAGAATAGAATAACTGACTCAGCTCAAGAGATCGGTCTGTTGTCAAACCTCAGCCTCACTAGCTCATTCAAAATAATTTTCACTATTGCCAAATCTCCTCTTATGGTTTTCTCCAAACATTATGCACACCCATGCCTAGCTGGATTCCCTTTCCTACCTACATGCACATGTGTGTTTATAAATGTATGTCTGCAAATGTTCGCTTATCAAGTCAAGGTGAACGTACGCCAATAAGCCTGCATGTATTACATGCATACGCATTCCTAGCTAGCTAAAATTCCCTGCATTCTCTCTCTCCTTCTCTCATCCTCCCCCCCCCCCCCCCCTTCCCAAAAGCATG

**>*HuDODA1-promoter***

AATAATAATAATAATAATAATAATATCTTATTATATACATTAGCGTGCATATACACATTCTTGCTTGTACTCTTAACAATGATATTTTTTTAGTTGGTTGCACTGTATTTTTGGGTGAGCTATCATATATATTGGTGAGCAATACTTTATGTTCATCTTATAAGGTGCTATGATATACCTTTTGGCAAGTTATGATATATTTGATAAGTCCTATTCATATGTGTTACATATGAATAGGTTGTCTCCATGTGACCAAAAGGTTACGGGTTCGAGCCGTGGAATTAGCCATTGACAAAGTCAGATTAGGCTGCTTACAATTCACCCCTTAGGGCGCGGCCCTTCTCCGAACCTTGCGTGAACGCGGGATGCTTGTACACCGGGTTGCCCTTTTTTTTTTTAATCATATGTACTACCTTTTTATAAAAGAGCGACCGTATAATATTTTGTTGAGGTTTGATATACATTTAGTGAGTTATGATATACATTTAGTGTGCTATGATGTGTATTTAGTAGCAGATGTTAGTAAGACAATATATATTGGTGAACCAAATAATAATAATAATAATAATAATAATAATAATAATAATAATAATAATAATAATAATTAATAACAATAATAATAATAACAACAATAACAATAACAATAACAATAAAAACAACATGTCATATTGTATACATTAGCAAGCACATATAGGCACACTCTTGTTTATGTTCTTAGCTATAATATTTTTTATTAGTGAGCTACACTATGTCTTTTGGTGAGCTGTGTTATAAATTTAGTAAGCAATACTTTATGTTCATTTGTTAGTTGCTATGATATATGTTTTGGTGAGTCATAATAAGTTTGATAAGTTTTATTCACATGTACTATGTATTTTTTAAGACACCAGTTGTATGATATTTTGATGCGTTATGATATACATTTAGTAAGCTACAATATATACTTAATGAGCAAACGTTAATGAGTTGTACCATATATGTTAGTGAGTTGAACGTATGTGTACATGTGTAGGTGTTAGTGAGTTAGATGGTATTTTTAGTAAGTGGAACCCAAGAGTACAAAAAATAGGCATTAGTGAGTTATACAATATTTTTAGTGTGTGAGTCACTACTCAAATTAATAACATAAAAGAAGGGATTAGACAATCTTCTTTAAAAAAAGAGAAAGAGTTTCTTTTATTAAAAAAAAAAAAAAGAGTTCACTTGAATATAAAAGGCTAAAGGGGAAAAAACACATTTTTTTAAAAAAAAGAAAGAGTTTAGTTCTATCAACAAGGGGGGAAAAAAGAAAGACTTCAGTTGAGTATAAAAGGTTAGGGGACGAATGATGCACAAATTAAAAAGGATGAAAAAAATATTAAAAAAAGAAACTAAATGCACATGTTGCAAATATCAAAGTATCAAACCACAGATCTTGTGGTGAGGGTAAAAGCAGCAAGCCATCAAGCCAATTGGCCTGTATTGGTATGATACTATGATGCAAAGAAACAAATTATTTACCTTACCACTGATCATGCCGGACGTACAATTCAAATTATACGCTCGGTTGTACAATACAAAAACCGAATGTTATCCCTTGTCGAAATCATAGATGGTTTTTCTTTTATTTTATTTGACTTGAATGGTAGACGTGGAGTTTTACTCATCTCTTGACTACCATGAGCCCATGCTTGAGTAGTTACTTTTTTGGGCCTCCATTTCTCTGCCCATCTATTCTTGGAAACGCATGACATCAATGATTAAAGGATGTGAAGGAGATGTGTTGGGCAGTTGTTTTTTATGTTTTTTTTTTTTGCCCCTAACTATGGTTCTGAAGTCTATTTAAGCAGAATGAGGCAGTGATGATCGTCATTAGCAATTGCACCAGCAGCACCAGAAGCAAAGATG
